# Providing TB and HIV outreach services to internally displaced populations in Northeast Nigeria: Results of a controlled intervention study

**DOI:** 10.1371/journal.pmed.1003218

**Published:** 2020-09-09

**Authors:** Suraj A. Abdullahi, Marina Smelyanskaya, Stephen John, Haruna I. Adamu, Emperor Ubochioma, Ishaya Kennedy, Fatima A. Abubakar, Haruna A. Ago, Robert Stevens, Jacob Creswell

**Affiliations:** 1 SUFABEL Community Development Initiative, Gombe, Nigeria; 2 Stop TB Partnership, Geneva, Switzerland; 3 Janna Health Foundation, Yola, Adamawa State, Nigeria; 4 World Health Organization, North East Zonal Office, Bauchi, Nigeria; 5 National TB, Leprosy & Buruli Ulcer Control Programme, Abuja, Nigeria; 6 Ministry of Health, Gombe, Nigeria; 7 Ministry of Health, Yola, Adamawa State, Nigeria; 8 Yobe State TB & Leprosy Control Programme, Damaturu, Nigeria; 9 Independent Consultant, Manchester, United Kingdom; Johns Hopkins University Bloomberg School of Public Health, UNITED STATES

## Abstract

**Background:**

A decade of Boko Haram insurgency brought conflict, mass displacement, and the destruction of basic infrastructure to Northeast Nigeria. Over 2 million internally displaced persons (IDPs) suffering from lack of basic hygienic conditions, malnutrition, and disease live in camps or are hosted by communities in the region, where the conflict has contributed to a massive destruction of health facilities. Infectious diseases like tuberculosis (TB) and HIV are especially difficult to address under such conditions, and IDPs are vulnerable to both. Although international investment supports some health interventions among IDPs, locally sourced solutions are lacking.

**Methods and findings:**

We evaluated the impact of an active case finding (ACF) intervention for TB and testing for HIV in IDP communities and provided linkages to treatment in 3 states in Northeast Nigeria: Adamawa, Gombe, and Yobe. The ACF was a component of a multistakeholder collaboration between government, civil society, and IDP community partners, which also included mapping of IDP populations and health services, supporting existing health facilities, developing a sample transport network, and organizing community outreach to support ACF. Between July 1, 2017, and June 30, 2018, ACF was conducted in 26 IDP camps and 963 host communities in 12 local government areas (LGAs) with another 12 LGAs serving as a control population. Outreach efforts resulted in 283,556 screening encounters. We screened 13,316 children and 270,239 adults including 150,303 (55.6%) adult women and 119,936 (44.4%) men. We tested 17,134 people for TB and 58,976 for HIV. We detected 1,423 people with TB and 874 people living with HIV. We linked 1,419 people to anti-TB treatment and 874 people with HIV to antiretroviral treatment sites. We evaluated additional TB cases notified and conducted comparative interrupted time series (ITS) analyses to assess the impact of ACF on TB case notifications. Through our efforts, bacteriologically confirmed TB notifications increased by 847 (45.1%) during the intervention period, with IDPs accounting for 46% of these notifications. The ITS analyses detected significant positive postintervention trend differences in TB notification rates between the intervention and control areas in all forms TB (incidence rate ratio [IRR] = 1.136 [1.072, 1.204]; *p* ≤ 0.001) and bacteriologically positive TB (IRR = 1.141 [1.058, 1.229]; *p* = 0.001). The TB prevalence (502 cases per 100,000 screening encounters) was 10 times the national notification rates and 2.3 times the estimated national incidence. Rates of HIV infection (1.8%) were higher than HIV prevalence estimates in the 3 states. Our study was limited by the nonrandom selection of LGAs. Furthermore, we did not use sensitive screening tools like chest X-ray and likely missed people with TB.

**Conclusions:**

In this study, we observed a burden of TB in IDP populations of Northeast Nigeria many times higher than national rates and HIV rates higher than state level estimates. The impact of the intervention showed that ACF can greatly increase TB case notifications. Engaging IDP communities, local governments, and civil society organizations is essential to ensuring the success of interventions targeting TB and HIV, and such approaches can provide sustained solutions to these and other health crises among vulnerable populations.

## Abstract 2

Suraj Adbullahi and colleagues assess tuberculosis and HIV case-finding among internally displaced persons in Nigeria.

## Author summary

**Why was this study done?:**

- A decade of Boko Haram insurgency in Northeast Nigeria displaced more than 2 million people and caused the decimation of basic health infrastructure.
- Internally displaced persons (IDPs) settle in camps but also move into host communities; in both settings, resources are minimal, nutrition is in short supply, and basic sanitation and access to healthcare may be lacking.
- These challenges, in addition to the stress of migration and Nigeria’s high HIV and tuberculosis (TB) burden, make IDPs especially vulnerable to these 2 highly linked infectious diseases, yet data and interventions to address this reality are scarce.

**What did the researchers do and find?:**

- We describe the results of a controlled intervention study on the impact on tuberculosis notifications and assessed HIV prevalence among IDPs in Northeast Nigeria from July 1, 2017, until June 30, 2018.
- With participation and support from local authorities, community-based organizations, IDP camp leaders, and community volunteers, we tested 17,134 people for TB and 58,976 people for HIV in Adamawa, Gombe, and Yobe State IDP camps and hosting communities, detecting 1,423 people with TB and 874 people living with HIV.
- In the intervention area, bacteriologically confirmed TB notifications increased by 45% compared with the year before, with IDPs accounting for 46% of such cases, while notifications decreased 6.3% in control areas.
- Among the IDP camp and host community populations, we found a TB burden 2.3 times the estimated national TB incidence and 10 times the national notification rates, whereas HIV prevalence among IDPs was above reported prevalence in the 3 states.

**What do these findings mean?:**

- Our results highlight the severe TB burden among IDPs in Northeast Nigeria, warranting concerted efforts to address TB in these populations.
- Our findings stress the importance of local, community-driven multistakeholder solutions designed to increase healthcare access for vulnerable populations, strengthen health systems, and address infectious diseases such as TB and HIV.
- The results of our intervention also demonstrate the need for expanding HIV services in IDP camps and in communities that host large numbers of IDPs.

## Introduction

A decade of Boko Haram insurgency in Northeast Nigeria has resulted in mass internal displacements in the Adamawa, Bauchi, Borno, Gombe, Taraba, and Yobe states. The International Organization for Migration (IOM) estimated over 2 million persons were internally displaced in Northeast Nigeria as of August 2019 [1].

Nigeria’s federal government collaborated with international and local partners to establish internally displaced person (IDP) camps in Northeast Nigeria; however, provision of health services in these camps has been limited, and the quality of these services has been questioned [2,3]. The conditions may be even worse in host communities, where 62% of IDPs have settled [4]. Residents of these communities—both IDPs and hosts—are reportedly experiencing resource depletion, shortages of food, lack of basic sanitation, and impeded access to basic health services [4,5].

A 2015 editorial in *The Lancet* warned that disruptions to basic infrastructure and health services in the Northeast region of Nigeria, the large numbers of people displaced, and living conditions in both formal and informal settlements where IDPs congregated would lead to disease outbreaks and setbacks to progress made against HIV, tuberculosis (TB), and malaria [6]. Multiple humanitarian agencies [5,7] and the Nigerian government have indeed reported critical malnutrition and disease outbreaks. Several cholera outbreaks [8] were reported, including a recent one in Adamawa in 2019 [9]. An outbreak of meningitis was recorded in the area in 2016, whereas malaria has been a persistent health problem among IDPs [10].

In 2017, only half of the individuals living with HIV in the Northeast states were receiving treatment, largely due to the closure of clinics able to provide antiretroviral treatment (ART) [10]. An estimated 1.4% of Nigeria’s total population, 1.9 million people, are living with HIV [11,12], making Nigeria second only to South Africa in the absolute number of people living with HIV (PLHIV) on the continent [13]. Nigeria also remains among the world’s highest TB burden countries, accounting annually for about 4% of the world’s incident TB cases with an estimated incidence rate of 219/100,000 people [14]. The struggle to contain the nation’s generalized TB and HIV epidemics may be attributable to the generally poor state of primary healthcare services [15]. These challenges are even more pronounced in the Northeast, where two thirds of health facilities were said to have been damaged by the conflict and were still not operational in 2018 [5].

Overcrowding, malnutrition, migration stresses, and the trauma of conflict [16,17] combine with Nigeria’s overall high TB burden to put IDPs, as well as the communities hosting them, at imminent risk for TB. Destruction of health services as a result of armed conflict in the region has contributed to the crisis [5,18]. Effective TB detection, prevention, and care depend on a functioning health system [19]. Although verbal screening for TB symptoms such as prolonged cough can happened anywhere, bacteriological diagnosis of TB must occur in a properly equipped laboratory. Samples from or individuals with presumptive TB must be transported to these labs. Delivering TB test results to highly vulnerable and mobile populations such as IDPs has to be rapid, and TB treatment has to commence immediately to prevent deaths and transmission in the community [17]. In addition to the availability of functioning health systems, TB detection and prevention depends on successful community engagement [19]. However, engaging IDP communities in TB screenings may be difficult because of persistent stigma, poor understanding about TB, and other challenges including a lack of trust in healthcare providers [20]. Addressing HIV, including prevention, testing, and treatment, is similarly complex, especially in populations who have been exposed to the trauma of conflict, sexual violence, and migration [21].

TB and HIV service delivery have specific requirements, but these are not significantly different from what may be needed to address other health disparities. Therefore, creating or rebuilding functional pathways for TB detection and care and HIV testing and treatment linkages can generate an opening to improve delivery of other health services [22,23]. Rebuilding health linkages in Northeast Nigeria for the thousands of IDPs and host community members requires engagement of these groups, collaboration between state governments and civil society, and restoration of the health system infrastructure destroyed by the conflict. This may only be possible through locally sourced solutions that are driven by local leaders, informed by the needs of at-risk populations and capacities of the local health systems, and supported by the engagement of all relevant stakeholders. Although international support has maintained a lifeline for IDPs and host communities, such locally based approaches have been lacking.

Here, we present the results of a public–private partnership approach delivered through a collaboration between state HIV and TB entities, 4 community-based organizations (CBOs), and community, tribal, IDP and religious leaders in Adamawa, Gombe, and Yobe states. The partnership focused on IDPs in camps and host communities, promoting an integrated service package involving active outreach for TB case finding and HIV testing, and support for management and care of the 2 conditions, as well as strengthening existing health facilities to help prevent disruptions in the provision of health services.

## Methods

This study followed the Transparent Reporting of Evaluations with Nonrandomized Designs (TREND) checklist for reporting standards of public health intervention evaluations involving nonrandomized designs (S1 TREND Checklist). The methodology followed the standard TB REACH monitoring and evaluation framework to determine the impact of TB case-finding interventions on notifications in an intervention area compared with historical and contemporary controls. The methodology uses project intervention data on the TB screening cascade, which feeds into official TB program notifications to document the burden of disease among different populations, the ability of different case-finding methods to detect people with TB, and finally, the impact on TB notifications [24]. The baseline validation including the project plan is included in S1 Text. Next, we detail both the approach to the intervention and the analysis used.

### Intervention area

The intervention took place between July 1, 2017, and June 30, 2018, in the Adamawa, Gombe, and Yobe states, which comprise 49 local government areas (LGAs) (21, 11, and 17 respectively). According to the latest census, which was conducted in Nigeria in 2006, the population of the 3 states at the time was 7,865,329 [25]. We estimated population growth of 2.8% per year, arriving at a 2017 population of 10,657,151 for the 3 states. IOM estimated that in these states, there were 276,061 IDPs in August 2017, of whom 54% were female, which is slightly higher than the national population proportions of 49%. Children under the age of 6 comprised 28% of the IDP population, while the national average is 19% [26].

**Fig 1:**
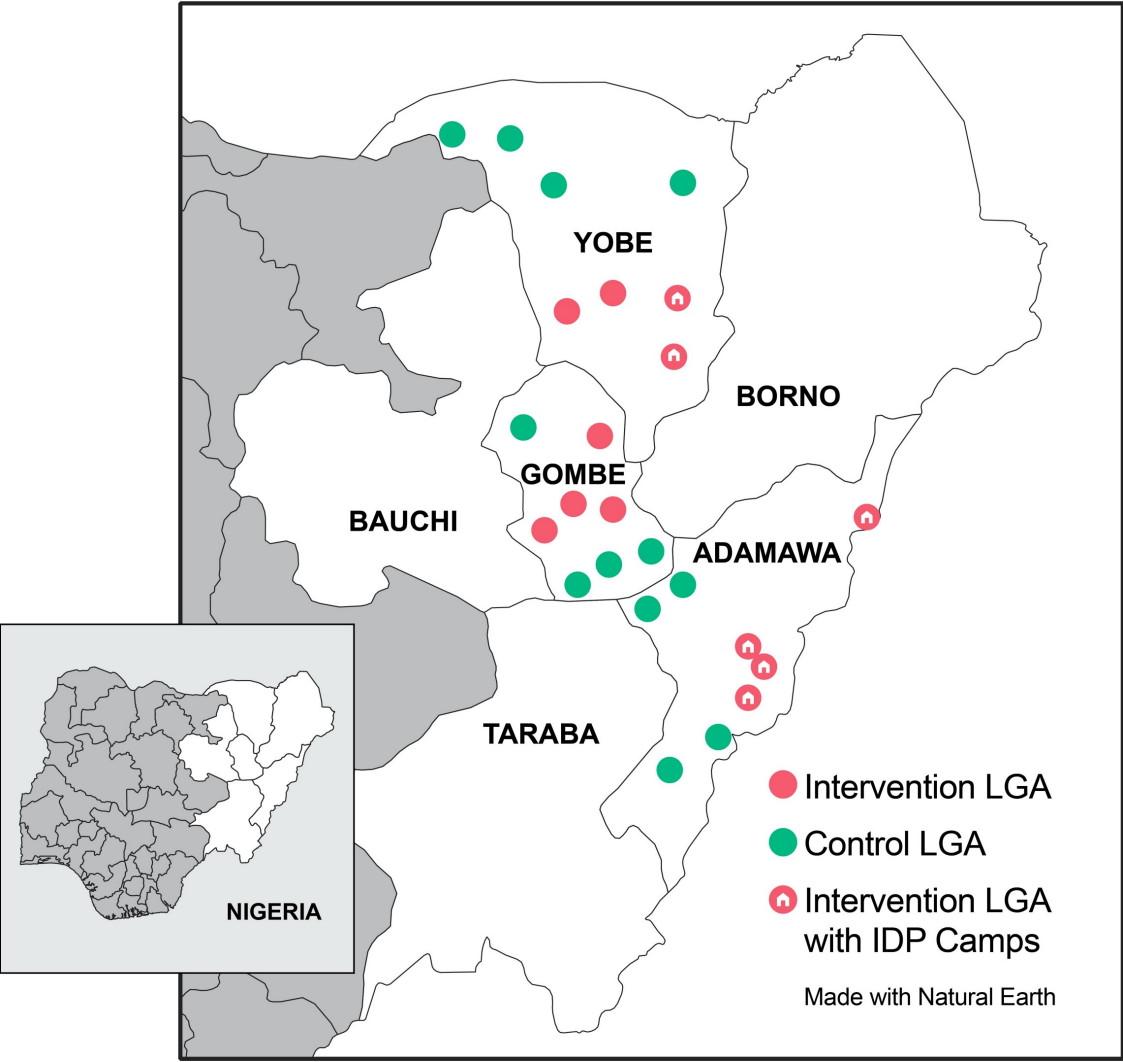
Map of intervention and control areas for IDP outreach activities. IDP, internally displaced person; LGA, local government area.

Within the 3 states, 12 LGAs (4 per state) were selected as a convenience sample for the intervention based on the number of IDPs in the LGAs and the presence of IDP camps and host communities to maximize potential impact. Adamawa and Yobe had IDP camps and host communities. For Gombe, selected LGAs reported having host communities with the largest number of IDPs in the state but had no IDP camps. Based on estimated population growth following the 2006 census, the population of the 12 intervention LGAs would have been 3,362,345 in 2017. We also selected 12 control LGAs with a combined estimated population of 2,461,374 in coordination with the local governments because they lacked active case-finding activities. See Fig 1 for a map of the intervention and control areas.

### Mapping IDP populations and host communities and available TB diagnostic and treatment facilities

At the time of our intervention, IOM site assessments were conducted in 34 IDP camps and camp-like structures in Adamawa and Yobe states (there were no camps in Gombe) [4]. We mapped and reconfirmed 26 IDP camp sites linked to health services in the 12 intervention LGAs. In the same 12 LGAs, we also mapped 962 IDP host community sites. Two of the 26 IDP camps had treatment facilities for TB, but none had diagnostic testing services, and none offered HIV testing, counseling, or treatment services.

In collaboration with state TB programs, HIV agencies, and 4 civil society organizations (CSOs) with experience in delivery of HIV and TB services in the 3 states (Janna Health Foundation, SUFABEL Community Development Initiative, Mallam Sidi Progressive Association and Taimako Community Development Initiative), we were able to map relevant health facilities in the 12 LGAs. There were 91 health facilities for diagnosis and treatment of TB cases but only 31 were functional in which diagnostic testing could be completed at the start of the project. Of the 31 functional sites, 12 had access to molecular testing with Xpert MTB/RIF (Xpert), whereas the 19 remaining used smear microscopy. There were 17 ART sites for the treatment of HIV in the same areas.

During the mapping, health facilities located in the proximity of IDP camps and host communities were noted, and healthcare workers (HCWs) were selected from these health facilities for training. These selected facilities provided treatment for TB cases diagnosed among the IDPs.

### Public–private partnerships for service provision

To facilitate smooth implementation, a state project team (SPT) was established in each of the states. Each SPT was led by the head of the State Agency for the Control of AIDS and included the State TB Control Officer, State Laboratory Quality Assurance Officer, and the Monitoring and Evaluation Officer from the implementing CBO, who served as the secretary. The SPT conducted a stakeholder analysis and identified key stakeholders for engagement. The following stakeholders were identified: National Emergency Management Agency (NEMA); State Emergency Management Agency (SEMA); camp commandants; camp medical directors and officials; humanitarian partners; Ministry of Health; State Primary Health Care Development agencies; State Agencies for the Control of AIDS; Ministries for local governments; Primary Health Authority of each LGA; and traditional, tribal, religious and host community leaders. The SPT developed advocacy kits and led advocacy visits to identified stakeholders to raise awareness on TB and HIV and facilitate acceptance of the intervention.

With guidance from the state TB program in each state, the implementing LGA TB supervisors, community leaders and camp managers, we selected IDPs from camps and host communities across the LGAs for training and conducting active TB case finding. Similarly, HCWs, including TB and laboratory staff, and medical officers, were identified and engaged for the intervention in each state. The community members and the HCWs were trained using the National TB and Leprosy Control Programme (NTBLCP) standardized training tools, and the State HIV Control Agencies also provided training on HIV counseling and testing. The training and engagement of lay community workforce continued throughout the intervention.

### Support to existing health facilities

The LGA authorities, in coordination with local TB programs, State Agencies for the Control of AIDS, and local CBOs, identified those facilities most in need of support. These facilities received a targeted package determined by the needs assessment. Support included solar panels and batteries for electricity and back-up power supply as well as minor infrastructure improvements, including roof renovation; replacement of furniture, doors, and windows; and in 1 case, purchasing a refrigerator for sample storage. Of the 12 GeneXpert sites where sputum samples were sent for diagnosis, 6 were provided with updates and support. The intervention also provided TB recording and reporting tools for use in the 31 functional treatment centers. Printed banners and information, education, and communication (IEC) materials to commemorate the World TB Day were provided as well.

This intervention ensured TB treatment services were also integrated into existing health clinics established by other organizations in the IDP camps. A sputum transportation component run by trained volunteers was also developed and linked to designated GeneXpert sites for diagnosis. At the outset of the intervention, staff of testing sites were notified about the likely increase in workload during screening and testing campaigns.

After observing that some of the strategically located health facilities had an inadequate health workforce, the CBOs appealed to the LGA’s Primary Health Care Authority, which resulted in the posting of additional HCWs, to 6 treatment centers serving people diagnosed with TB in IDP camps and host communities in Yobe and Gombe States. These salaries were paid for with public and private funds.

### Outreach interventions for TB and HIV

Two types of outreach efforts were carried out. The first intervention was based in IDP camps, and the second targeted the host communities. In each LGA, monthly plans were made for IDP camp screenings, and each camp was screened about 6 times. Community screenings were conducted biweekly in each LGA. Additional outreach screenings were conducted during times when many new arrivals came to the IDP camps or communities.

Outreach screenings in IDP camps and host communities were preceded by a sensitization visit targeting relevant stakeholders and coordinated with partners so as not to disturb other activities. Community mobilizers were identified within the camps or host communities based on language skills, and knowledge of the community. Supplies and commodities (HIV rapid testing kits, sputum collection containers, screening tools, IEC materials, multivitamin, deworming medication, etc.) were secured by the state HIV and TB programs. Furthermore, laboratory staff at the laboratory sites were informed prior to the outreach to facilitate better management of sample flow from the targeted areas.

In camps, people were asked to congregate in specified meetings areas, where TB screening and HIV counseling would be preceded by health education delivered with the aid of the IEC materials. Key contents of the IEC materials covered the cause, transmission, signs, symptoms, treatment, and prevention of TB and HIV. This was followed by verbal informed consent and symptom screening of IDPs for prolonged cough (2 weeks or more) to identify people with presumptive TB for sputum collection and the offering of HIV testing to all individuals. In addition, tent-to-tent screening was carried out by the HCWs from the CBOs and camp representatives, who visited all tents at the camp to reach people who could not or chose not to participate in the group screenings. For some camp events, visiting tents was not possible because of fears about infiltration from Boko Haram members. In these cases, only congregate area screening was conducted.

During the host community outreach, the LGA supervisor allocated groups of 3 to 5 HCWs, each including at least 1 female to ensure access to female-led households. In the host communities, new metal-roofed homes were built to accommodate the inflow of IDPs. These, and other homes housing IDPs, were mapped in each community in consultation with community leaders. The host community screening was done systematically with an attempt to visit every house, taking a street at a time to avoid missing houses during the process. If household members were absent, a repeat visit was planned. Because not all homes could be visited every time, and some people were always out of the home, not all could be screened. A community guide led the screening team to each house, where household members were enumerated. Verbal TB symptom screening was conducted, and HIV counseling and testing were offered to all individuals, as in the camp outreach.

Once people with presumptive TB were identified through these outreach efforts, TB diagnosis followed standard NTBLCP guidelines. For all outreach activities, people with presumptive TB were registered and had one spot sputum sample collected, labeled, and stored in sputum boxes with ice packs. The sputum boxes were transported to the nearest GeneXpert laboratory at the end of each screening day. People with positive Xpert results (both drug sensitive and rifampicin resistant TB) were linked to treatment and notified as bacteriologically positive (B+) cases. For those IDPs who consented verbally, HIV testing was done using serial testing with Alere Determine. Those who tested positive for HIV on the rapid test were actively referred to the nearest secondary health facility for confirmation, in line with the National HIV guidelines [27]. All people diagnosed with HIV were actively linked to the nearest ART site for treatment, care, and support. People were offered Levamisole (a deworming medication) as well as multivitamins as an incentive for participation in the screening. Outside of outreach days, individuals experiencing TB symptoms were referred to the IDP camp HCW for TB screening coordinated with the nearest TB treatment facility.

### Result retrieval and linkage to treatment

**Fig 2:**
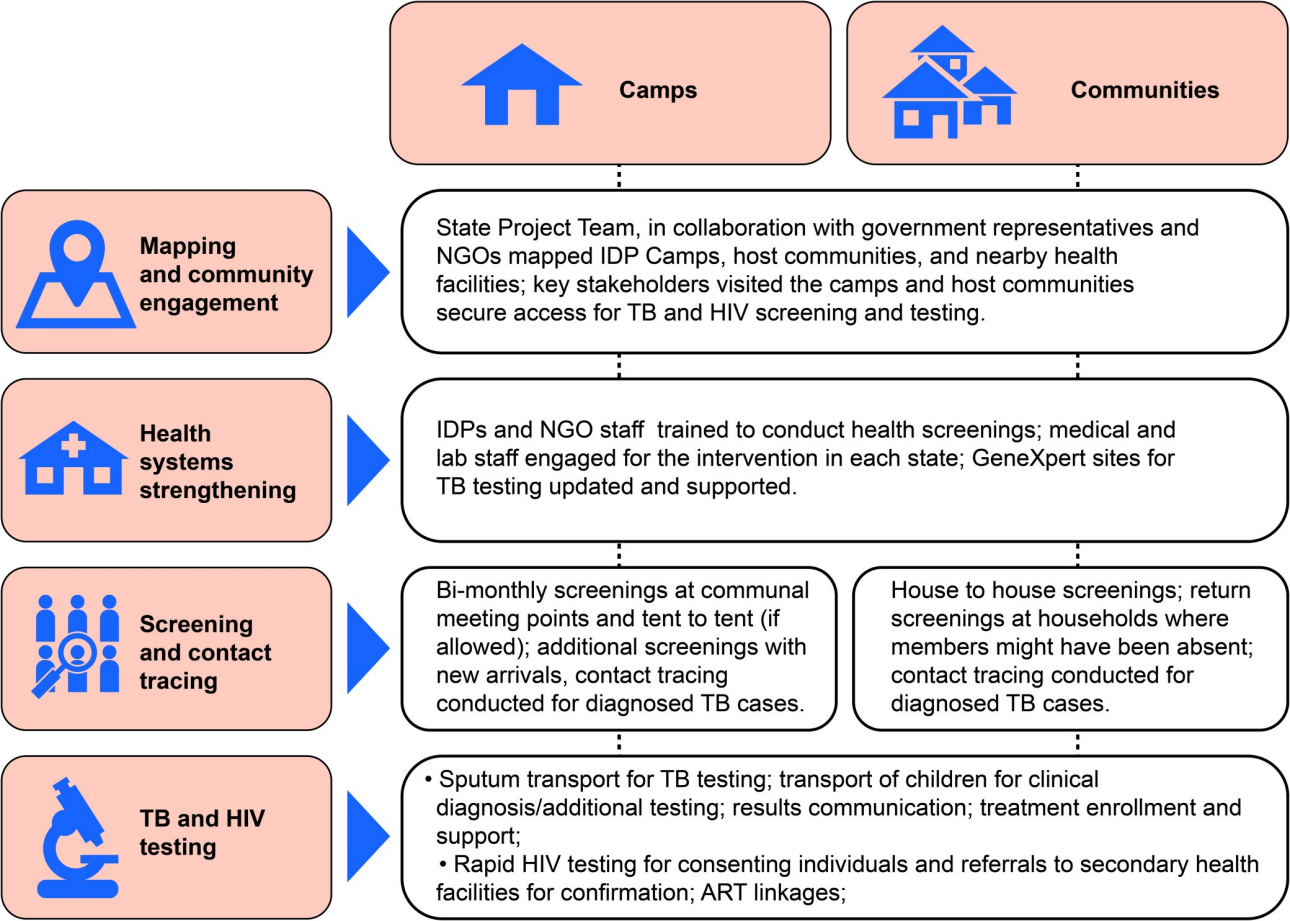
Key steps necessary for outreach and support interventions in IDP camps and hosting communities in Northeast Nigeria. ART, antiretroviral therapy; IDP, internally displaced persons; NGO, nongovernmental organization; TB, tuberculosis.

Results from GeneXpert testing sites were retrieved after each outreach episode and returned to the IDP camps and host communities by either the LGA TB supervisors or designated volunteers. Individuals with B+ TB were enrolled at the nearest treatment center; individuals who were confirmed as HIV-positive at the secondary health facility were also linked to ART sites. Individuals with TB symptoms, but who were not bacteriologically confirmed, were referred to medical officers at the nearest secondary health facility for further clinical evaluation. Transportation support was offered for those needing urgent care. However, we were unable to document the numbers of people receiving evaluation for clinically diagnosed TB. IDPs were identified in all recording tools by inserting the acronyms “IDP” in the “remarks” column in the standard TB registers and at the top corner on individual patient cards. This marking facilitated the identification of IDPs during the quarterly state TB program review meetings. All household contacts of people with confirmed TB, including children under 6 years of age, were investigated again by the local health staff or the LGA TB supervisor through home visits for verbal screening. Because children require a more comprehensive clinical evaluation for diagnosis, transportation to the nearest secondary health facility for additional screening was arranged by local CBOs. When TB disease was ruled out among child household contacts less than 6 years of age, TB preventive therapy (TPT) was offered. Fig 2 outlines the key steps for implementing TB and HIV screening in IDP camps and communities.

### Data analysis

Individuals diagnosed with TB through the intervention were disaggregated from state case notification data during quarterly NTP review meetings. All individuals diagnosed with TB notified among IDPs during the study period were included in this analysis. Information on age, sex, laboratory results, TB classification, TB/HIV coinfection, and treatment outcome was extracted. Standardized definitions for these variables based on NTBLCP guidelines were used. TB case notification and laboratory data were also retrospectively collected from 2014–2017. Changes in TB case notification trends for B+ and all forms of TB (B+ and clinically diagnosed) were calculated from the difference between case notifications during baseline and intervention periods. TB REACH interventions generally use simple regression analysis to document the change in TB notifications, but to address the potential autocorrelation of TB notification data and to evaluate the change in notification trajectory after the intervention, we chose a different analysis. To assess the validity of changes in notification, we conducted comparative interrupted time series (ITS) analyses of aggregate quarterly TB case notification rates for the intervention and control areas. The ITS analyses employed segmented methods applied to marginal log-linear Poisson regression models using the generalized estimating equation (GEE) approach. We tested for serial autocorrelation using the Cumby–Huizinga test with a cutoff of *p* < 0.1 and specified the model based on the lowest quasi-likelihood information criterion values. Statistical analyses were performed on Stata version 13 (StataCorp, https://www.stata.com/). Approval to implement the intervention was obtained from the Adamawa, Gombe, and Yobe State Ministries of Health, as well as the State Agencies for the Control of AIDS and the National TB and Leprosy Control Programme. All individuals had consented prior to TB and HIV testing.

## Results

Key stakeholders, including the state HIV agencies and TB programs, LGA supervisors, CBO leaders, State Ministry of Health Officials, and IDP camp officials, held a series of planning meetings and sensitization visits before outreach efforts were undertaken from May 2017 until August 2017. A total of 96 advocacy visits were jointly conducted across the 12 LGAs to enlist the support of key stakeholders. A total of 180 volunteers (95 male and 85 female) from the IDP communities were formally trained on outreach and health education, screening, and patient support. A total of 66 HCWs (28 male and 38 female) from treatment sites and 15 medical officers (12 male and 3 female) from secondary health facilities were retrained on TB detection, treatment, and management. Staff from the CBOs were also trained on active TB case finding, diagnosis, linkage to treatment, care, and support. Orientation on HIV counseling and testing was provided by staff from the State Agencies for the Control of AIDS during monthly meetings with volunteers.

From July 1, 2017, to June 30, 2018, we conducted active outreach for TB screening and HIV testing in 234 of the 963 host communities and 165 separate episodes of TB screening in 26 IDP Camps in the 3 states. The total number of IDPs living in the camps fluctuated daily, so providing an accurate camp population is impossible. However, available records from the camps revealed that camp populations ranged from 1,211 to 14,645 across the 3 states. A summary of the screening efforts across the 3 states is presented in Table 1.

**Table 1:** Results of active case finding for TB in IDP camps and host communities by state.

	Adamawa	Gombe	Yobe	Total
Number screening encounters (% of total screened)	138,272 (48.8%)	111,296 (39.3%)	33,988 (12.0%)	283,556
Number of people identified with TB symptoms (% of screened)	9,791 (7.1%)	5,447 (4.9%)	4,414 (13.0%)	19,652 (6.9%)
Number tested for TB (% of those with symptoms)	8,198 (83.7%)	5,035 (92.4%)	3,901 (88.4%)	17,134 (87.2%)
Number of people with laboratory-confirmed TB (% of tested)	637 (7.8%)	194 (3.9%)	426 (10.9%)	1,257 (7.3%)
Number of people diagnosed with TB all forms (per 100,000 screening encounters)	729 (527)	240 (216)	454 (1,336)	1,423 (502)
Number of all forms TB patients started on treatment (% of diagnosed)	726 (99.6%)	239 (100%)	454 (100%)	1,419 (99.7%)

IDP, internally displaced person; TB, tuberculosis.

Overall, we counted 283,556 verbal screening for TB encounters among the IDP population. Almost half of the screening (48.8%) was conducted in Adamawa, whereas 12.0% came from Yobe. Because of repeat visits and anonymized screening efforts, we can only report the number of encounters rather than individuals screened. Overall, we counted 270,239 screening encounters among adults and 13,316 among children with 150,303 (55.6%) adult screening encounters among women and 119,936 (44.4%) among men. Once TB symptoms were identified, individuals were provided ID numbers and enumerated, so other than screening numbers, the rest of the data provided here indicates the numbers of individuals. The proportion of people with TB symptoms was 6.9% (*n* = 19,652) among all screened, and we were able to collect sputum samples for testing from 17,134 of them (87.2%). Overall, the TB rates per 100,000 screening encounters was 502, but this varied between 216 in Gombe and 1,336 in Yobe.

Table 2 presents the TB screening cascade for the outreach efforts. Through verbal screening, 19,652 individuals reported 1 or more TB symptom and were eligible to submit a sputum sample, and 17,134 had their samples tested by GeneXpert. Overall, we identified 1,257 people for whom B+ TB was confirmed, including 442 in IDP camps and 815 in host communities. All but 2 initiated treatment. In addition, 166 people were diagnosed clinically so that overall, our outreach efforts identified 1,423 people with TB and started 1,419 of them on treatment.

**Table 2:** Results of active case finding for TB in IDP camps and host communities.

Indicator	Screening at IDP Camp	Screening in Host Communities	Total
Number of screening encounters	90,738	192,818	283,556
Number of people with presumptive TB	7,259	15,425	19,652
Number of people tested	6,315	10,819	17,134
Number of people with laboratory-confirmed TB	442	815	1,257
Number of people clinically diagnosed	156	10	166
Total all forms TB detected	598	825	1,423
Number of Bac+ TB patients started on treatment	442	813	1,255
Number of all forms TB patients started on treatment	596	823	1,419
All forms yield per 100,000 screening	659	428	502
All forms number needed to screen	152	234	199

IDP, internally displaced person; TB, tuberculosis.

The total all forms TB yield from screening was 502/per 100,000 screening encounters (1,423 cases among 283,556 screening encounters). The host community screening yielded 428 cases per 100,000 screening encounters, whereas the IDP camp screening yielded 659 cases per 100,000 screening encounters. The number needed to screen (screening/cases) to identify a person with TB was 199 overall, varying from 152 in camps to 234 in host communities.

Of all TB cases notified by this project, Xpert testing identified 58 (3.5%) cases of rifampicin resistance including 56 adults (28 females) and 2 children. Active follow-up of household contacts yielded an additional 38 B+ and 18 clinically diagnosed cases from examining 2,025 contacts (2.7%). A total of 821 children were found eligible for TPT; 552 (67.3%) received TPT (6 months course of Isoniazid) out of which 409 (74%) completed the course (data not shown).

### HIV testing results

HIV testing results can be found in Table 3. A total of 58,976 IDPs were provided counseling and testing for HIV using rapid tests. The majority of testing was among adults (51,309), including 27,304 females and 24,005 males, whereas 7,667 (13.0%) of people tested were children under 15. We identified 874 (1.5%) people to be HIV+. Among them, 530 (60.6%) were females, and 68 (7.8%) were children under the age of 15. The adult prevalence in the sample was 1.6% (1.1% among males and 1.9% among females) and 0.9% was calculated in children under the age of 15. Persons found to be HIV-positive were linked to ART sites. TB cases notified from the IDP camps and host communities through outreach efforts also received HIV counseling and testing at the treatment facilities; 398 (28%) of the 1,419 all forms of TB cases notified had TB/HIV coinfection. They were all started on ART. We did not document the impact of our intervention on the number of new HIV cases registered in the intervention LGAs.

**Table 3:** HIV testing results among IDP camps and host communities in Northeast Nigeria.

	Adults					Children < 15		Total
	Males		Females		Total	*n*	%	
	*n*	%	*n*	%				
Tested	24,005	40.7%	27,304	46.3%	51,309	7,667	13.0%	58,976
HIV+	276	31.6%	530	60.6%	806	68	7.8%	874
HIV prevalence	1.1%		1.9%		1.6%	0.9%		1.5%

IDP, internally displaced person.

### Impact on TB notifications

The number of people tested for TB in laboratories increased dramatically during the intervention period as shown in Table 4. In the 4 quarters prior to the intervention (July 2016 through June 2017), the diagnostic facilities tested a total of 15,996 people, detecting TB in 1,806. During the outreach to IDPs, the number of tests increased to 28,321, and 2,726 people were detected with TB (including the 1,257 by the intervention), an increase of 77% in testing and 51% in diagnosed cases from baseline, whereas the positivity rate decreased from 11.3% in the baseline period to 9.6% during the intervention.

**Table 4:** Laboratory testing and results for the Adamawa, Gombe, and Yobe states during baseline and intervention periods.

Period	Quarter	Number of People Tested	Number of People Tested Positive (B+)	Positive Yield	% Change in Cases Detected^a^
Baseline	Q3 2016	2,802	361	12.9%	
	Q4 2016	4,276	487	11.4%	
	Q1 2017	4,864	489	10.1%	
	Q2 2017	4,054	469	11.6%	
Total Baseline		15,996	1,806	11.3%	
Intervention	Q3 2017	6,500	599	9.2%	65.9%
	Q4 2017	6,894	645	9.4%	36.9%
	Q1 2018	7,940	779	9.8%	59.3%
	Q2 2018	6,987	703	10.1%	49.9%
Total Intervention		28,321	2,726	9.6%	50.9%

^a^ % change in TB cases detected compares intervention quarter to corresponding baseline quarter full year.

In the 4 quarters preceding the intervention, the 12 intervention LGAs notified 1,878 B+ cases and 3,348 all forms cases for estimated notification rate of 56 out of 100,000 population for B+ cases and 91 out of 100,000 for all forms using the 3.36 million population estimate. During the active outreach intervention, the same LGAs notified 2,725 B+ cases and 3,900 all forms cases, corresponding to 81 and 116 out of 100,000 notification rates, respectively, and an increase of 16.5% in all forms cases and 45.1% among those with B+ results. IDPs comprised 46% of all B+ notifications and 36% of all forms cases. In the control areas, we observed a 9.2% decline in B+ (722 from 795) and 6.3% decline in all forms notifications (974 from 1,040) during the intervention (Table 5).

**Table 5:** Change in TB case notifications by study area, Northeast Nigeria.

	Cumulative Notifications			Additionality	
	Baseline Period	Intervention Period		# Cases	% Change
All forms TB					
Intervention area	3,348	3,900		552	16.5%
Control area	1,040	974		−66	−6.3%
B+ TB					
Intervention area	1,878	2,725		847	45.1%
Control area	795	722		−73	−9.2%

B+, bacteriologically confirmed; TB, tuberculosis.

**Fig 3:**
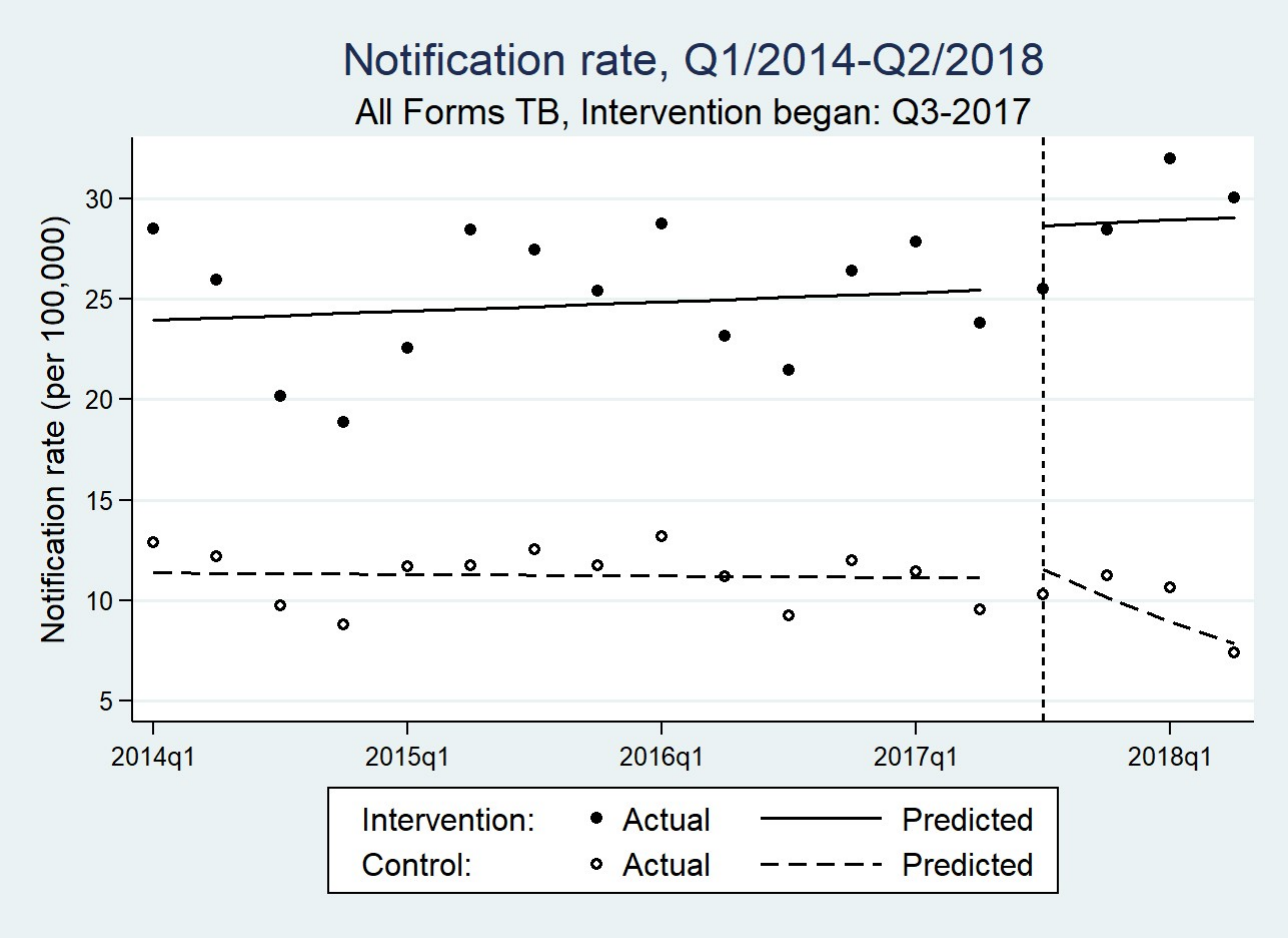
Quarterly all forms TB notifications rates before and after IDP screening efforts in intervention and control areas of Northeast Nigeria. IDP, internally displaced person; Q, quarter; TB, tuberculosis.

**Fig 4:**
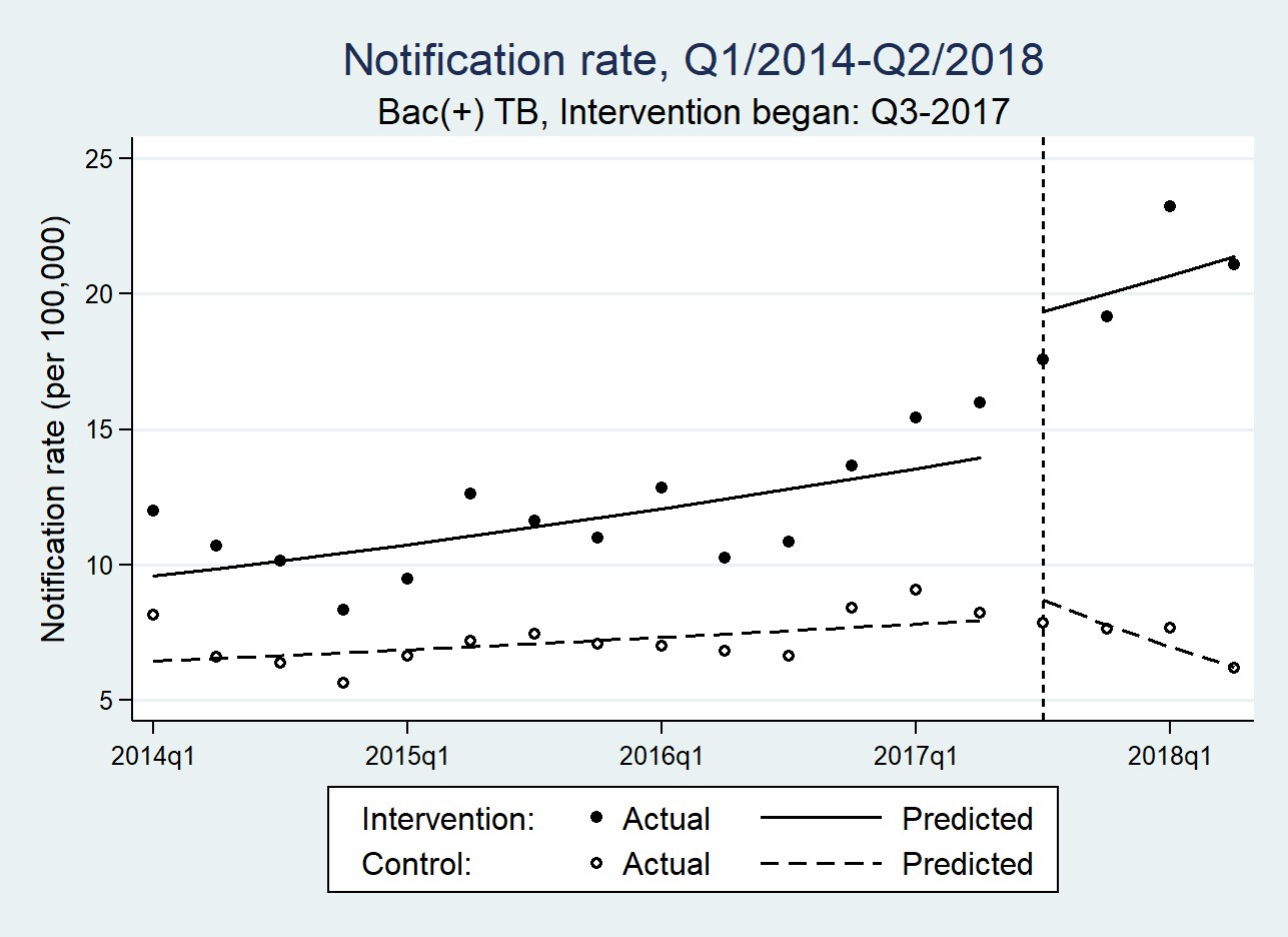
Quarterly bacteriologically positive TB notifications rates before and after IDP screening efforts in intervention and control areas of Northeast Nigeria. IDP, internally displaced person; Q, quarter; TB, tuberculosis.

The time series data consisted of 36 quarterly counts of aggregate treatment notifications for each B+ and all forms, balanced between intervention and control LGAs. The quarterly median all forms TB notifications was 54 (IQR range: 34–96) in the intervention area and 19 (IQR: 21–29) in the control area. The ITS analyses results are presented in Fig 3, Fig 4, and Table 6. In the postimplementation period, there was a significant trend difference between the intervention and control areas in all forms TB (incidence rate ratio [IRR][β7] = 1.136 [1.072, 1.204]; *p* ≤ 0.001) and B+ TB notification rates (IRR [β7] = 1.141 [1.058, 1.229]; *p* = 0.001). There was also a difference in the preintervention trends (*p* = 0.041).

**Table 6:** Comparative ITS analysis model parameters^a^ of population-standardized quarterly notification rates of all forms and bacteriologically confirmed TB cases for intervention versus control areas.

	Intervention Versus Control		
	IRR^b^	95% CI	*p*-value^c^
All Forms TB			
Baseline rate^d^ (*β*_*0*_)	11.380	[10.933, 11.844]	<0.001
Pre-intervention trend, control (*β*_*1*_)	0.998	[0.993, 1.004]	0.526
Postintervention step change, control (*β*_*2*_)	1.040	[0.949, 1.139]	0.403
Postintervention trend, control (*β*_*3*_)	0.881	[0.836, 0.928]	<0.001
Difference in baseline (*β*_*4*_)	2.105	[2.010, 2.205]	<0.001
Difference in pre-intervention trends (*β*_*5*_)	1.006	[1.000, 1.013]	0.041
Difference in postintervention step change (*β*_*6*_)	1.078	[0.971, 1.195]	0.159
Difference in postintervention trends (*β*_*7*_)	1.136	[1.072, 1.204]	<0.001
Bacteriologically confirmed TB			
Baseline rate^d^ (*β*_*0*_)	6.438	[6.074, 6.824]	<0.001
Pre-intervention trend, control (*β*_*1*_)	1.016	[1.009, 1.024]	<0.001
Postintervention step change, control (*β*_*2*_)	1.078	[0.949, 1.225]	0.247
Postintervention trend, control (*β*_*3*_)	0.881	[0.824, 0.941]	<0.001
Difference in baseline (*β*_*4*_)	1.486	[1.385, 1.595]	<0.001
Difference in pre-intervention trends (*β*_*5*_)	1.013	[1.004, 1.022]	0.005
Difference in postintervention step change (*β*_*6*_)	1.250	[1.078, 1.450]	0.003
Difference in postintervention trends (*β*_*7*_)	1.141	[1.058, 1.229]	0.001

^a^The parameters were obtained for a segmented regression model with the following structure: $Y_{t}=\beta_{0}+\beta_{1}T_{t}+\beta_{2}X_{t}+\beta_{3}X_{t}T_{t}+\beta_{4}Z+\beta_{5}ZT_{t}+\beta_{6}ZX_{t}+\beta_{6}ZX_{t}T_{t}+\epsilon_{t}$. Here, Y_t_ is the outcome measure along time t; T_t_ is the quarterly time counter; X_t_ indicates pre- and postintervention periods, Z denotes the intervention cohort, and ZT_t_, ZX_t_, and ZX_t_T_t_ are interaction terms. β_0_ to β_3_ relate to the control group as follows: β_0_, intercept; β_1_, pre-intervention trend; β_2_, postintervention step change; β_3_, postintervention trend. β_4_ to β_7_ represent differences between the control and intervention districts: β_4_, difference in baseline intercepts; β_5_, difference in pre-intervention trends; β_6_, difference in postintervention step changes; β_7_, difference in postintervention trend.

^b^IRR based on log-linear GEE Poisson regression with correlation structures determined by the Cumby–Huizinga test and quasi-information criteria.

^c^Wald test.

^d^The baseline rate denotes case notification rates per quarter.

GEE, generalized estimating equation; IRR, incidence rate ratio; ITS, interrupted time series; TB, tuberculosis.

## Discussion

The impacts of crisis and migration on the increased risk and transmission of TB are well documented [16,17,28,29], and our results confirm that IDP communities in Boko Haram–affected areas of Nigeria have not been spared this risk. The burden of TB among IDPs screened we found was more than twice the estimated incidence in Nigeria and 10 times the national notification rates [14]. Nigeria’s TB notifications have remained mostly flat for the last decade, and with case detection rate of 24%, many people with TB remain undiagnosed [14]. Our intervention suggests that concerted efforts to engage key populations at risk for TB could help with advancing TB notifications that are currently below national [30] and global targets [31]. By focusing on a subset of an IDP population in Northeast Nigeria, we were able to improve notification rates of bacteriologically confirmed TB by almost 50%. This increase in TB notifications observed between the baseline and intervention periods was made possible through dedicated and repeated verbal symptom screening and the significant increase in testing, coupled with follow-up contact investigation. As the global health community has come to realize the urgency of increasing the numbers of people diagnosed and treated for TB [32,33], streamlined and improved access to laboratory testing must be achieved to reach these goals. Accordingly, developing effective and rapid sample transportation networks to increase coverage and improved laboratory infrastructure as described in our methods is essential. Our results also indicate that despite increasing the number of diagnostic tests conducted by more than 50% compared with before the intervention, the yield of the tests remained quite high, similar to passive case finding [34]. Other active case-finding interventions have reported much lower yields [35,36], suggesting that in Northeast Nigeria, greater gains in TB detection can be accomplished by additional screening. Increased access to additional tests such as chest X-ray can help improve the screening cascade to detect more people to test and improve clinical diagnosis [37].

Research on HIV in IDPs is scarce. A study in the Democratic Republic of the Congo demonstrated that women in IDP camps had HIV prevalence that was significantly higher than that of women in neighboring communities [38]. In 2004, Paul Spiegel—United Nations High Commissioner for Refugees at the time—warned of the risks of stigmatizing IDP and refugee communities as those that contribute to the spread of HIV [39]. Since then, it has been established that mass rapes and survival transaction sex affecting IDP and refugee women in recent conflicts [21,40–42] may impact HIV prevalence in these vulnerable populations but also affect national HIV prevalence and incidence [43]. However, because a systematic review of interventions that target antiretroviral care for HIV in displaced populations has documented adequate adherence in IDPs, while also profiling the need for multistakeholder engagement and preparedness, it is clear that HIV test-and-treat interventions with IDPs can be successful, leading to outcomes matching those of stable settings [44]. Our results show that among the unrepresentative sample of adult IDPs in Northeast Nigeria, HIV prevalence was 1.6% whereas the estimated prevalence of HIV among adults in all 3 states was between 0.4%–1.3% [11]. In our sample, females comprised 46.3% of those tested for HIV but accounted for 60.6% of all HIV cases, resulting in a HIV prevalence among adult women of 1.9%, which was higher than that among men (1.1%). This is consistent with national data [45] and indicates additional vulnerability to HIV among IDP women. Our results also documented an 0.9% prevalence of HIV among IDP children under the age of 15 while the national rate was 0.2% [45]. Considering persistent reports of sexual violence in the Boko Haram–affected areas and coercion into transactional sex among female IDPs in camps and informal settlements [43], emerging evidence of challenges in delivering prevention of mother-to-child transmission services in Nigeria’s IDP camps [46], and the fact that we only tested of a small sample of IDPs, these results should be a source for concern.

Our intervention demonstrates that concerted efforts to create partnership between government, civil society, and international humanitarian stakeholders, the involvement of affected communities and providing support to health infrastructure can result in successful delivery of health services to vulnerable IDP populations even in challenging operating environments. Our efforts to build trusting relationships with IDPs and communities that host them can serve as a valuable lesson for future interventions intending to operate in similar environments [47], in which disparities may impact the health and development of entire nations [48,49]. The failure to consult with local communities and a lack of transparency in controlling outbreaks are largely to blame for overwhelming distrust of international health aid in Haiti [50,51]. Similarly, lack of community engagement and ignorance of diverse community needs and practices have been repeatedly highlighted as factors contributing to the length and severity of recent Ebola epidemics and outbreaks [52–55].

We placed tribal chiefs and informal camp leaders in IDP camps and community and religious leaders in host communities on an equal playing field with camp commandants (who are also senior military personnel), as well as with humanitarian organizations, emergency management agencies (state and federal levels), and health personnel. This was crucial for securing trust and establishing a transparent flow of information between all stakeholders and for ensuring full community buy-in and engagement. In some communities, there were dire challenges in achieving acceptance of volunteers. Young community members who volunteered to conduct educational campaigns and lead TB screening and HIV testing efforts were met with distrust and even violent resistance. This was quickly identified by community and religious leaders as resulting from persistent fears in the community over Boko Haram’s use of young and adolescent children for suicide bombings. To increase community trust, volunteers were provided with identification cards and reflector aprons bearing the names of the CBOs recognized in the communities. Although simple, this and other solutions were crucial in delivering health services to vulnerable populations with high levels of postconflict trauma.

Although we documented very high levels of TB among the IDPs in our intervention area, our sample was not randomly selected and cannot be considered representative. Likewise, the selection of intervention LGAs was done with the intention of maximizing impact on case finding, and the results of the ITS showed significant differences in pre-intervention trends, which limits the usefulness of the control comparator. However, we feel there is clear evidence of a large increase in the intervention LGAs, and we selected the control districts based on lack of other interventions before the project began. The movement of people in and across the 3 states where we worked complicated our data management efforts and calculations of impact and outcomes. Because of these challenges, we cannot make conclusive statements about TB incidence or HIV prevalence in IDP communities in Northeast Nigeria. However, our results document a heavy burden of disease in these groups and the acute need for tailored service delivery models. Although we did not utilize chest X-ray, this addition would allow even more people with TB to be detected because of X-ray’s higher sensitivity compared to verbal screening and its ability to support more comprehensive clinical diagnosis [56]. Future interventions may take this into consideration.

IDPs in camps and host communities lack access to basic health services and are disproportionately affected by TB and HIV. Our intervention shows that concerted efforts at community engagement and a multisectorial collaboration can improve delivery of critical health services even in communities impacted by crisis and displacement. In addition to community buy-in, increased access to high-quality diagnostics at health facilities, a functional sample transportation system, and grassroots mobilization for outreach and contact tracing were key parts of the intervention. It is crucial for relevant government agencies, communities, and other key stakeholders in Northeast Nigeria to continue generating locally sourced solutions to resolve TB, HIV, and other health disparities among IDPs in the region. These efforts will contribute to strengthening health systems and creating resilient communities.

## Supporting information

S1 Text Baseline validation and summary of intervention plans. (DOCX)

S1 TREND Checklist TREND, Transparent Reporting of Evaluations with Nonrandomized Designs. (DOCX)

S1 Data TB notification data used in interrupted time series analysis and additionality calculations. TB, tuberculosis.
(XLSX)

## Abbreviations

ART: antiretroviral treatment
CBO: community-based organization
HCW: healthcare worker
IDP: internally displaced person
IEC: information, education, and communication
IOM: International Organization for Migration
; IRR: incidence rate ratio
LGA: local government area
NTBLCP: National TB and Leprosy Control Programme
PLHIV: people living with HIV
TB: tuberculosis
TPT: TB-preventative therapy

## References

[1] International Organization for Migration (IOM) [Internet]. Displacement Tracking Matrix, International Organization of Migration, Nigeria [cited 2019 Sep 16]. Available from: https://displacement.iom.int/nigeria

[2] Ekezie W, Timmons S, Myles P, Siebert P, Bains M, Pritchard C. An audit of healthcare provision in internally displaced population camps in Nigeria. J Public Health. 2019 9 30;41(3):583–592. 10.1093/pubmed/fdy141 30137460

[3] Taylor-Robinson SD, Oleribe O. Famine and disease in Nigerian refugee camps for internally displaced peoples: a sad reflection of our times. QJM Int J Med. 2016 12;109(12):831–834. 10.1093/qjmed/hcw171 27795293 PMC5903602

[4] International Organization for Migration (IOM) [Internet]. Nigeria, displacement tracking matrix. Round 18 report. 2017 [cited 2019 Sep 16]. Available from: https://displacement.iom.int/reports/nigeria-%E2%80%94-displacement-report-18-august-2017

[5] UN Office for the Coordination of Humanitarian Affairs (UNOCHA). Nigeria: 2019 humanitarian needs overview. 2019 [cited 2019 Sep 16] Available from: https://reliefweb.int/report/nigeria/nigeria-2019-humanitarian-needs-overview

[6] Omole O, Welye H, Abimbola S. Boko Haram insurgency: implications for public health. Lancet. 2015;385:941 10.1016/S0140-6736(15)60207-0 25747581

[7] Food Security Cluster [Internet]. FICHE report: Cadre Harmonisé for Identification of Risk Areas and Vulnerable Populations in Sixteen (16) States and the Federal Capital Territory (FCT) of Nigeria. 2018 Oct [cited 2019 Sep 20]. Available from: https://fscluster.org/nigeria/document/fiche-report-cadre-harmonise

[8] Nigeria Centre for Disease Control [Internet]. Disease situation reports. [cited 2019 Sep 18]. Available from: https://ncdc.gov.ng/diseases/sitreps

[9] Borno State launches first Malaria Operational Plan, reawakens fight against malaria [Internet]. In: WHO | Regional Office for Africa. [cited 2019 Sep 18]. Available from: https://www.afro.who.int/news/borno-state-launches-first-malaria-operational-plan-reawakens-fight-against-malaria

[10] ACAPS [Internet]. Thematic report –24 May 2017, Nigeria, health in the Northeast. 2017 May [2019 Sep 20]. Available from: https://www.acaps.org/special-report/nigeria-health-northeast

[11] Nigeria prevalence rate [Internet]. National Agency for the Control of AIDS (NACA) Nigeria. [cited 2019 Oct 2]. Available from: https://naca.gov.ng/nigeria-prevalence-rate/

[12] UNAIDS [Internet]. New survey results indicate that Nigeria has an HIV prevalence of 1.4%. [cited 2019 Oct 2]. Available from: https://www.unaids.org/en/resources/presscentre/pressreleaseandstatementarchive/2019/march/20190314_nigeria

[13] UNAIDS [Internet]. Number of people living with HIV. In: AIDSinfo. [cited 5 May 2020]. Available from: https://aidsinfo.unaids.org/

[14] World Health Organization [Internet]. Global Tuberculosis report 2018. Geneva, Switzerland: WHO; 2018 [cited 2019 Oct 2]. Available from: http://www.who.int/tb/publications/global_report/en/

[15] Aregbeshola BS, Khan SM. Primary health care in Nigeria: 24 years after Olikoye Ransome-Kuti’s leadership. Front Public Health. 2017;5 10.3389/fpubh.2017.00048 28349050 PMC5346888

[16] Kronfol NM, Mansour Z. Tuberculosis and migration: a review. East Mediterr Health J. 2013;19: 739–748. 24975360

[17] Dhavan P, Dias HM, Creswell J, Weil D. An overview of tuberculosis and migration. Int J Tuberc Lung Dis. 2017;21: 610–623. 10.5588/ijtld.16.0917 28482955

[18] Eboreime E, Obi FA. How Boko Haram is devastating health services in North-East Nigeria. In: The Conversation [Internet]. [cited 2019 Oct 3]. Available from: http://theconversation.com/how-boko-haram-is-devastating-health-services-in-north-east-nigeria-65751

[19] World Health Organization [Internet]. Implementing the End TB Strategy: the essentials. 2015 [cited 2019 Oct 2]. Available from: https://www.who.int/tb/publications/2015/end_tb_essential.pdf?ua=1

[20] Stop TB Partnership [Internet]. Key populations brief: mobile populations 2016 [cited 2019 Oct 2]. Available from: http://stoptb.org/assets/documents/resources/publications/acsm/KP_Mobile_Spreads.pdf

[21] Marsh M, Purdin S, Navani S. Addressing sexual violence in humanitarian emergencies. Glob Public Health. 2006;1: 133–146. 10.1080/17441690600652787 19153902

[22] World Health Organization. Contributing to health system strengthening: Guiding principles for national tuberculosis programmes. 2008 [cited 2019 Oct 2]. Available from: https://www.who.int/healthsystems/Stop_TB_HSS_policy_paper_EN.pdf 24921118

[23] Justman JE, Koblavi-Deme S, Tanuri A, Goldberg A, Gonzalez LF, Gwynn CR. Developing Laboratory Systems and Infrastructure for HIV Scale-Up: A Tool for Health Systems Strengthening in Resource-Limited Settings. JAIDS J Acquir Immune Defic Syndr. 2009;52:S30 10.1097/QAI.0b013e3181bbc9f5 19858935

[24] Blok L, Creswell J, Stevens R, Brouwer M, Ramis O, Weil O, et al A pragmatic approach to measuring, monitoring and evaluating interventions for improved tuberculosis case detection. Int Health. 2014;6: 181–188. 10.1093/inthealth/ihu055 25100402 PMC4153747

[25] State population, 2006 [Internet]. Nigeria data portal. [cited 2019 Oct 3]. Available from: http://nigeria.opendataforafrica.org//ifpbxbd/state-population-2006

[26] Naitonal Population Commission. 2006 Population and Housing Census, Federal Republic of Nigeria [Internet]. 2017 [cited 2019 Oct 3]. Available from: https://web.archive.org/web/20170120201645/ http://www.population.gov.ng/images/Priority%20table%20Vol%204.pdf

[27] National guidelines for HIV prevention treatment and care–Nigeria [Internet]. National AIDS and STI’s Control Programme. Federal Ministry of Health, Nigeria; 2016 [cited 2019 Oct 3]. Available from: http://apps.who.int/medicinedocs/documents/s23252en/s23252en.pdf

[28] Ködmön C, Zucs P, van der Werf MJ. Migration-related tuberculosis: epidemiology and characteristics of tuberculosis cases originating outside the European Union and European Economic Area, 2007 to 2013. Euro Surveill Bull Eur Sur Mal Transm Eur Commun Dis Bull. 2016;21(12). 10.2807/1560-7917.ES.2016.21.12.30164 27039665

[29] Kimbrough W, Saliba V, Dahab M, Haskew C, Checchi F. The burden of tuberculosis in crisis-affected populations: a systematic review. Lancet Infect Dis. 2012;12:950–965. 10.1016/S1473-3099(12)70225-6 23174381

[30] The national strategic plan for tuberculosis control 2015–2020 [Internet]. National Tuberculosis and Leprosy Control Programme, Department of Public Health, Federal Ministry of Health, Nigeria; 2014 [cited 2019 Oct 3]. Available from: https://www.medbox.org/nigeria-the-national-strategic-plan-for-tuberculosis-control-2015-2020/download.pdf

[31] Stop TB Partnership [Internet]. TB diagnosis and treatment targets. 2018 [cited 2019 Oct 3]. Available from: http://www.stoptb.org/assets/documents/global/advocacy/unhlm/1.%20UNHLM%20on%20TB%20-%20TB%20Country%20Targets.pdf

[32] Joint initiative “FIND. TREAT. ALL. #ENDTB.” In: WHO. [cited 2019 Oct 4]. Available from: http://www.who.int/tb/joint-initiative/en/

[33] Stop TB and Global Fund deepen cooperation to find missing cases of TB [Internet]. [cited 4 Oct 2019]. Available from: https://www.theglobalfund.org/en/news/2017-12-18-stop-tb-and-global-fund-deepen-cooperation-to-find-missing-cases-of-tb/

[34] Challenge TB: annual report year 4 [Internet]. United States Agency for International Development (USAID); 2019 [cited 3 Oct 2019]. Available from: https://www.challengetb.org/reportfiles/Challenge_TB_Year_4_Annual_Report.pdf

[35] Corbett EL, Bandason T, Duong T, Dauya E, Makamure B, Churchyard GJ, et al Comparison of two active case-finding strategies for community-based diagnosis of symptomatic smear-positive tuberculosis and control of infectious tuberculosis in Harare, Zimbabwe (DETECTB): a cluster-randomised trial. Lancet. 2010;376: 1244–1253. 10.1016/S0140-6736(10)61425-0 20923715 PMC2956882

[36] Yassin MA, Datiko DG, Tulloch O, Markos P, Aschalew M, Shargie EB, et al Innovative community-based approaches doubled tuberculosis case notification and improve treatment outcome in Southern Ethiopia. PLoS ONE. 2013;8: e63174 10.1371/journal.pone.0063174 23723975 PMC3664633

[37] van’t Hoog AH, Meme HK, Laserson KF, Agaya JA, Muchiri BG, Githui WA, et al Screening strategies for tuberculosis prevalence surveys: the value of chest radiography and symptoms. PLoS ONE. 2012;7: e38691 10.1371/journal.pone.0038691 22792158 PMC3391193

[38] Kim AA, Malele F, Kaiser R, Mama N, Kinkela T, Mantshumba J-C, et al HIV infection among internally displaced women and women residing in river populations along the Congo River, Democratic Republic of Congo. AIDS Behav. 2009;13: 914–920. 10.1007/s10461-009-9536-z 19319674

[39] Spiegel PB. HIV/AIDS among conflict-affected and displaced populations: dispelling myths and taking action. Disasters. 2004;28: 322–339. 10.1111/j.0361-3666.2004.00261.x 15344944

[40] Omba JK. Sexual violence in the Democratic Republic of Congo: impact on public health? Med Trop Rev Corps Sante Colon. 2008;68: 576–578. 19639818

[41] Patel S, Schechter MT, Sewankambo NK, Atim S, Kiwanuka N, Spittal PM. Lost in transition: HIV prevalence and correlates of infection among young people living in post-emergency phase transit camps in Gulu District, Northern Uganda. PLoS ONE. 2014;9: e89786–e89786. 10.1371/journal.pone.0089786 24587034 PMC3938506

[42] Women in displacement camps in Nigeria resort to transactional sex for survival | Africa Renewal [Internet]. [cited 2020 May 5]. Available from: https://www.un.org/africarenewal/news/women-displacement-camps-nigeria-resort-transactional-sex-survival

[43] Supervie V, Halima Y, Blower S. Assessing the impact of mass rape on the incidence of HIV in conflict-affected countries. AIDS Lond Engl. 2010;24: 2841–2847. 10.1097/QAD.0b013e32833fed78 20859191 PMC2978669

[44] Griffiths K, Ford N. Provision of antiretroviral care to displaced populations in humanitarian settings: a systematic review. Med Confl Surviv. 2013;29: 198–215. 10.1080/13623699.2013.813108 24133930

[45] The Government of Nigeria. NAIIS preliminary findings, March 2019 [Internet]. 2019 [cited 2020 May 5]. Available from: https://www.naiis.ng/resource/factsheet/NAIIS%20PA%20NATIONAL%20FACTSHEET%20FINAL.pdf

[46] Nsikan A, Sunkanmi F, Peter D, Joseph J, Emmanuel O, Paul Y, et al PMTCT Service Uptake among Pregnant Women in 3 Internally Displace Persons Camps in Borno State Northeast Nigeria. J Adv Med Med Res. 2020;32(4):72–77.

[47] UNHCR. Nigeria situation 2017 [Internet]. 2017 [cited 2019 Oct 3]. Available from: https://www.unhcr.org/597704b87.pdf

[48] United Nations Development Programme. National human development report 2018 [Internet]. Achieving human development in North East Nigeria. Nigeria; 2018 [cited 2019 Oct 3]. Available from: http://hdr.undp.org/sites/default/files/hdr_2018_nigeria_finalfinalx3.pdf

[49] Awojobi O. The socio-economic implications of Boko Haram insurgency in the North-East of Nigeria. Int J Innov Sci Res. 2014;11: 144–150.

[50] Biquet J-M. Haiti: between emergency and reconstruction. An inadequate response. Int Dev Policy. 2013;4 3:129–135. 10.4000/poldev.1600

[51] Pham PN, Gibbons N, Vinck P. The United Nations material assistance to survivors of cholera in Haiti: consulting survivors and rebuilding trust. PLoS Curr. 2017;9 10.1371/currents.dis.1b01af244fe3d76d6a7013e2f1e3944d 29188126 PMC5693334

[52] West Africa’s Ebola epidemic is out of control, but never had to happen [Internet]. O’Neill Instititue; 2014 Aug [cited 2019 Oct 3]. Briefing paper no. 9. Available from: https://oneill.law.georgetown.edu/media/Briefing9Gostin_WestAfricasEbolaEpidemicisOutofControlButNeverHadtoHappen.pdf

[53] The Ebola response in West Africa: exposing the politics and culture of international aid. In: ODI [Internet]. [cited 2019 Oct 3]. Available from: https://www.odi.org/publications/9956-ebola-response-west-africa-exposing-politics-culture-international-aid

[54] World Health Organization. Ebola virus disease: Democratic Republic of the Congo. External situation report 61 [Internet]. 2019 10 [cited 2020 May 5]. Available from: https://reliefweb.int/sites/reliefweb.int/files/resources/SITREP_EVD_DRC_20190929-eng.pdf

[55] Thiam S, Delamou A, Camara S, Carter J, Lama EK, Ndiaye B, et al Challenges in controlling the Ebola outbreak in two prefectures in Guinea: why did communities continue to resist? Pan Afr Med J. 2015;22 10.11694/pamj.supp.2015.22.1.6626 26740850 PMC4695515

[56] Onozaki I, Law I, Sismanidis C, Zignol M, Glaziou P, Floyd K. National tuberculosis prevalence surveys in Asia, 1990–2012: an overview of results and lessons learned. Trop Med Int Health TM IH. 2015;20: 1128–1145. 10.1111/tmi.12534 25943163

