# Supplementary material for: Providing TB and HIV outreach services to internally displaced populations in Northeast Nigeria: Results of a controlled intervention study
Source: PLoS Med. 2020 Sep 9;17(9):e1003218. doi: 10.1371/journal.pmed.1003218 (PMC7480873; doi:10.1371/journal.pmed.1003218)
Supplement: S1 Text — (DOCX) [file pmed.1003218.s001.docx]

**BASELINE VALIDATION REVIEW**

**(WAVE 5)**

| Grant & Review Details:  Country: Nigeria  Grant Title:  Integrated Provider Initiated TB and HIV Services among Internally Displaced Persons in Northeastern Nigeria.  Name of Grantee Organisation: Gombe State Agency for the Control of AIDS GomSaca  Name of Grantee Project Director: Kwami Abdulkarim Suraj  Project Short Code: W5_NGA_CDT1_GomSACA  Type of project: Type 1 Type 2  Type 3  Name of M&E Reviewer: Osahon Ogbeiwi  Date of completion of review: 6^th^ June 2017 |
| --- |

BACKGROUND INFORMATION**^[[1]](#footnote-1)^**

Context

The project is located in three of the six States of the Northeastern geopolitical zone of Nigeria; these are Adamawa, Gombe and Yobe States. The zone covers a land area of 280,419 km2, about a third of the total area of the country. Besides the shared similar geographical, socio-economic, ethnographic, health situations, the States in the zone are the worst affected by the ongoing terrorist insurgency of the notorious Boko Haram group and military response of the Nigerian Federal Government. This has led to the largest civil crisis in Nigeria since the Biafran war in the 1960s, displacing millions of the civilian population from their natural homes into emergency humanitarian refugee camps and settlements scattered in many host communities. Most of these camps are located in 12 Local Government Areas located in the 3 States targeted by this project. In addition to the problem of insecurity, the Boko Haram scourge has damaged virtually every social infrastructure in the northeast, especially it caused a depreciation of available health systems and disease control services, which is considered least developed in the IDP settlements. At the height of terrorist scourge, thousands of IDPs arrived weekly into the humanitarian camps, mainly in these three States: 56% of them were children, 53% females and 7% elderly aged above 60. The camps suffered from inadequate basic amenities, poor sanitation, poor nutrition and inadequate accommodation that resulted in overcrowding and high risk of TB transmission. Health care is provided in tents, with limited TB and HIV services.

Following the national TB prevalence survey completed in 2012, the country’s TB prevalence rate was estimated to be 524 per 100,000 population and a projected incidence rate of 338 per 100,000. Up to 40% of TB patients found were asymptomatic and therefore did not seek treatment and are missed from the TB notifications reported through the national TB programme. Based on the national estimate, and the population of the 12 LGAs being 3,362,345, the estimated prevalence of TB in the project area is as high as 17,618 of all forms, with about 10% in IDP camps. However, much less is notified in the routine NTP reports for the area. For example, by the end of 2015 in the targeted 12 LGAs, 2,362 notifications of all forms of TB were reported, which was 7.5 times less than the survey-based estimate. A total of 1253 (53%) of them were smear / bacteriological positive, the sex ratio (M:F) was 2:1, and the age group with highest notifications being older adults aged 35-54 years. The notification rates were 70.24 per 100,000 population for all forms and 37.26 for bacteriologically positive patients. The overall treatment success rate was 89% with a range from 85% to 95%.

Up to 175,000 people live with HIV in the 3 States, with the HIV prevalence of 3.5% higher than the national average of 3.2%. The 12 LGAs targeted by the project has 91 DOTS centres and 31 AFB sputum microscopy centres available as health facilities for TB control. There are GeneXpert machines in 12 of the DOTS centres. For HIV, there are 17 comprehensive ART centres, in addition to the basic HIV services provided in all health facilities. TB service delivery in the project 12 LGAs is plagued by a high percentage of asymptomatic TB cases who do not seek care, weak collaborative and referral linkages and treatment success rate that is below the NTP standard of 95%. In addition, there is lack of TB management capacity in the humanitarian health care facilities in IDP camps and very few DOTS centres with GeneXpert diagnostic technology.

A past TB REACH project targeted the nomadic population in Adamawa State and was titled, ‘Innovative Delivery of TB care to Nigeria’s Nomadic and Koma People.’ A total of 1310 bacteriological positive patients were detected out of 96,376 nomads screened. The state-wide impact was seen in a 112% increase in the number of presumptive TB tested microscopically and 49.5% increase in the number of new smear positive notifications.

Project Description

The project is an innovative active case finding (ACF) intervention to increase the detection of TB and HIV among internally displaced persons (IDPs) and their linkage with the treatment services of the national TB and HIV control programmes. It is a type 1 project with an approved TB REACH grant at the level of proof of concept, which if successfully implemented should have enough sustainability to be scaled up in other and wider areas with IDP camps in the North-Eastern zone. The project targets the IDP population in 12 LGAs in 3 States, 4 LGAs each in Adamawa, Gombe and Yobe States. While the principal recipient of the TB REACH grant is Gombe State HIV/AIDS Control Agency, the grantee will partner with the State TB Control programmes in the three States in the implementation and monitoring of the project activities in their respective State. The primary overall aim of the project is to detect additional notifications of TB patients among IDPs in the targeted 12 LGAs as the measurable impact of the project at its end. The secondary purpose is to conduct an operational research to estimate the prevalence of TB, HIV and TB/HIV coinfection among IDPs in North-Eastern Nigeria. The specific shorter-term objectives to be monitored quarterly are the cascaded results of symptomatic screening of IDPs in the camps and host communities to identify TB presumptive cases and bacteriologically testing the latter for confirmation of sputum smear positive patients using the GeneXpert technology.

The project will use two screening strategies and a phased approach to achieve these goals. The strategies include a total population house-to-house screening visits to the IDP caps and mass TB/HIV screening and HCT outreaches in the camps and host communities. The field screening will be the collaborative responsibility of 4 Community Based Organisations (CBOs), 2 in Gombe State and 1 each in Adamawa and Yobe States. 180 Community Volunteers (CVs), 15 per LGA will be engaged by the 4 CBOs. Implementation of the key activities of the project rests solely on the shoulders of the CVs, as they will conduct the verbal screening for TB presumptive cases, collect and transport sputum samples to the GeneXpert locations, retrieve results and link confirmed patients to the DOTS centres of the State TB Control programme for treatment and followup. The project will refer bacteriologically negative TB presumptive cases to clinicians in health facilities for clinical diagnosis and subsequent linkage to DOTS centres for treatment. The CBOs will be responsible for creating awareness and mobilising camps and communities for the screening visits and outreaches. They will directly supervise the CVs working in their designated LGAs. Both CVs and CBOs will be supervised periodically in each LGA by the LGA TB supervisors (1 per LGA), who is the designated personnel for the implementation of the State TB Control Programme at the LGA level and collation of TB notification data from all DOTS centres in his or her jurisdiction.

The project’s data will be aligned with the data management procedures of the State TB programme that rests heavily on the LGA TB supervisors, who registers confirmed TB patients in the DOTS centres monthly, reports TB case notifications, collated from all DOTS centres in each LGA, quarterly and submits quarterly statistical reports to the State level TB programme managers during the quarterly LGA TBL Programme Review meetings. Data of new TB notifications from the targeted IDP camps and host communities, the 12 LGAs constituting the evaluation and control populations will be retrieved from the LGA TBL supervisors during this meetings by the project’s designated M&E officers in each of the 3 States.

Purpose of Baseline Validation Review

It is the purpose of this baseline validation report to validate the baseline data provided in the grant documents by the grantee, and to assess the monitoring and evaluation (M&E) framework for the project. This BV review with its specific intervention-level process indicators lays the foundation for all subsequent M&E with an overall purpose to report the project’s results and demonstrate additional TB notifications, intervention-level outcomes based on the project indicators and other impacts of the project.

A map showing the 3 States, 12 LGAs in the evaluation population, and 12 LGAs in the control area is placed in Annex 1.

## SUMMARY OF REVIEWER’S REVIEW

The baseline data is:

Valid but different from the data in the full proposal

- The baseline target data represent adjusted figures compared to the target data in the proposal, as the grantee attempted to make them more realistic for the project. Similarly, the number of CBOs and CVs have been adjusted downward and upward respectively.

Recommendations for quarterly reports

a

Scale up and advocacy, learning from implementation

Key points:

- The project plans to use community-based stakeholders of the IDP camps and host communities to execute its advocacy visits to relevant government authorities at State and LGA levels. These activities need to be sustained throughout the project implementation, if the expected advocacy results will be achieved at the end of the project.
- With the network of partnerships and organisational capacity for advocacy, there is a high chance of sustainability post TB REACH grant.

Populations and targets

Key points:

- With the relatively small size of the target population, compared to the evaluation population, there is a high risk of dilution of the project yield during the quarterly and terminal reviews. However the grantee believes the high risk of TB among IDPs reported by a KNCV survey may help the project still achieve its project.

Notifications in evaluation and control population

Key points:

- The population sizes of the evaluation and control LGAs are considered comparable and are estimates for 2016 projected from the 2006 National census using 2.8% annual growth rate.
- No data was reported for one of the LGAs in the evaluation population (Gujba) through the 12 quarters from 2014 to 2016 due to intensified activities of the Boko Harram insurgents which resulted to a total displacement of the entire LGA population.
- The TB case notification figures in the control population (CP) generally reflect lower case detection than in the evaluation population (EP), and possibly suggest a marked difference between EP and CP.
- The high %Bac+ among AF notifications in some CP LGAs and the level of disparity from the national value gives concern the quality of notifications data. According to grantee’s explanation, the high B+/AF% in the CPs is due to poor access to medical officers for clinical diagnosis of TB in these LGAs and the improved access to Gx machines especially among HIV clients. Thus a higher number of B+ cases are diagnosed and registered by HWs in these LGAs
- Some of the EP and CP LGAs are contiguous. Is it possible project found cases could be registered in the CP? Is it possible that CP residents might be drawn in to the project? These questions need to be addressed during the RP1 stage.

Activities.

Key points:

- About half of activities are in the preparatory stage which will be completed by the end of June. The implementation stage will start in the first week of July in a State-by-State phased strategy.
- Grantee revised the number of CBOs from 6 to 4 and that of CVs from 120 to 180 with 15 per LGA. Despite this, each CV may still screen about 3,170 persons or 264 persons per month.
- The workplan requires specific implementation dates for each activity.
- Advocacy activities should not be restricted to the preparation stage alone, but continued all through the project term. A dissemination plan should be included in the project workplan

Process indicators

Key points:

- The project plans to use two main interventions – a House-two-House screening and a HCT community screening outreach that includes contact tracing of index TB patients.
- In trying to be realistic, the grantee has adjusted the TB notification targets
- In an ideal project situation, all presumptive TB persons should be tested, and all confirmed TB patients, whether Bac+ or AF should be registered for anti-TB treatment. The process indicators bear targets that suggest this will not be the case in this project. According to the grantee, it is the project aim to screen all presumptives and place all diagnosed TB cases on treatment, but there are unforeseen circumstances that may not guarantee 100%; thus the deficit.

External Factors

Key points:

- The main risk factor is the unpredictable relocation, migration or movement of IDPs in and out of the evaluation LGAs or the outright closure of the IDP camps. A mitigation plan should be considered.

As mitigation according to the grantee, the government will maintain at least 1 IDP Camp in each of the 4 LGAs to ease service provision. Smaller camps will be closed or merged with big camps.

Data quality assessment

Key points:

- Plans to use existing DQA processes of the NTP in each State. More project specific approaches to ensure the quality of data is required.

In response, the grantee plan to include data validation in the field in their routine supportive supervision, monthly CBO and CVs meetings and quarterly NTP meetings in each State. Data from the intervention will be collated at the quarterly NTP meetings at the State after going through the data validation process.

Follow up for project patients on treatment

Key points:

- Plans to use existing follow-up processes of the NTP in each State. More project specific approaches to ensure the quality of data is required.

Grantee explained they will use the same supervisory structures as outline above to follow-up project patients placed on treatment.

Operational research

Key points:

- Research protocol not yet prepared. This will be prepared after completion of the BV reporting process.

## DETAILED ANALYSIS OF THE PROPOSAL AND DEVELOPMENT OF the M&E FRAMEWORK

# Main interventions

Table 1 shows the two interventions that will be used by the project for actuive case finding (CF). The house-to-house verbal screening of IDP camps and host communities will be done by community volunteers, and while the community-based organisations will execute the mass community HCT screening. While both interventions will actively search for TB presumptive cases using a checklist of TB symptoms, for screening with GeneXpert sputum tests, the mass community screening outreach will additionally conduct HIV counselling and testing (HCT) to detect persons with HIV infection and TB/HIV co-infection in the IDP camps and host communities. The total number needed to be screen according to the grantee is 195,989, with 70% expected to be screened using the house-to-house strategy. The daignostic algorithm is illustrated in appendix 1.

Table 1: Interventions

| Intervention | Classification |
| --- | --- |
| 1. House to House Verbal Screening of IDPs | By whom: Community Volunteers  Where: Community-based set-up in 4 LGAs each in Adawama, Yobe and Gombe States (total 12 LGAs)  Population screened: Migrants/Refugees/IDPs  Other strategies supporting CF: Household visits,  First test: GeneXperts sputum test  Improved diagnostics: |
| 2. Mass Community HCT Screening Outreach | By whom: Community Based Organisations  Where: Community-based set-up in 4 LGAs each in Adawama, Yobe and Gombe States (total 12 LGAs)  Population screened: Migrants/Refugees/IDPs  Other strategies supporting CF: Contact tracing, HIV couselling and testing, community advocacy and mobilisation  First test: HIV Test  Improved diagnostics: GeneXperts sputum test |

Are the proposed interventions part the National Strategic Plan of the NTP?

Yes  No  Unknown/partially/(other)

Do the proposal’s interventions include any of the following (if a major intervention, then it should be described in the table above):

Preventive treatment

Treatment outcome

# Scale up and advocacy, learning from implementation

**Advocacy Processes:**

1. Grantee Strategic Advocacy Planning.

According to the response of the grantee, four levels of advocacy have been planned:

- National-NPT manager, WHO
- State level- SEMA, SPHCDA and commissioners of health in the 3 states, SACAs
- LGA - PHC coordinators, facility staff
- Community level- Camp commandant, community leaders

At the national level, the grantee has kept the national coordinator of the NTP in the loop of the project application and approval processes. In line with the sustainability plan in the project proposal, the project will be implemented with a strong cooperation and partnership with Government structures involved in the national TB control at all tiers, national, State and LGA. In particular, the grantee reported that key stakeholders have been identified at the community level, who will be mobilised into advocacy teams that will plan and embark on visits to State-level legislators and ministries of health and finance to solicit for local funding and integration of the project activities and budget into existing Government structures for TB control. There is also the plan to empower the advocacy teams with regular information of the results and progress of the project, so they have the necessary knowledge to communicate the advocacy message to authorities at the State and LGA levels. Also important is the plan to mobilise at the community level the existing ward committees in the project LGAs during the official inauguration of the project and involve them in the local advocacy during the project term.

(This box will be visible by the grantee)

| I believe the advocacy is compatible with the sustainability in the grant proposal. The planned advocacy activities are valid and culturally appropriate. The grantee plan to exercise their advocacy activities using mainly community level stakeholders, with no hint of the extent of involvement of Gombe SACA as the responsible agency. While this bottom-up advocacy approach will build a sense of ownership of the project in the host communities, my concern is challenge of how this advocacy work community stakeholders and ward committees will be organised effectively in the 12 LGAs and 3 States that will be covered by this project.  In response, the grantee agrees that GomSACA as well as the 2 other SACAs will carry out the high-level advocacies targeting political office-holders like the Commissioners and Governors. GomSACA, the lead agency will coordinate all other advocacies across the 3 States. |
| --- |

1. Political Support

It is intended that the intervention of targeted screening for TB among IDPs will be included into the State TB programme budget. The project will identify new partnerships with other government agencies and form operational alliances with non-government partners already working in the camps and groups of IDPs. GF and TB Challenge are not currently present in the project area. There is likelihood of continuation of HCT Intervention by HIV funders due the drive toward achieving 90-90-90 target of HIV testing services.

Additionally, according to the grantee, the project has mapped out key stakeholders at all levels in each of the States in addition to the NTP, NACA and NASCP. GomSACA will lead the advocacy visits to the NTP, NACA and NASCP at the onset of the project and whenever the need arises. This will ensure smooth flow of project implementation and could have a positive impact on sustainability and scalability within the country. GomSACA will lead the State Project Teams headed by SACAs in each of the States to conduct a high level advocacy targeting the identified policy makers and politicians. This will ensure the reflection of the intervention in the health budget and facilitate approvals as direct funding to the State TB Programmes and SACAs for strengthening of TB and HIV control among IDPs.

(This box will be visible by the grantee)

| The proposed plan will make a positive political impact on project implementation and sustainability when GomSACA being a government agency secures the support of government at all levels for the project. |
| --- |

1. Funding Stability

Table 2 shows the plan for long-term post TB REACH funding of the project activities which will rest on three government organisations at the State and regional level. The budget for NTP activities in the State is currently provided by direct government spending on human and material resources and Global Fund grants, which will continue beyond the TB REACH grant. As stated in the grant proposal, the intervention funding will be included into the State TB control programme budgets, and the activities will be integrated into existing government structures at both State and LGA levels.

The SACA organisations in the three States will also continue to provide local funding for the HCT services in the project LGAs beyond the TB REACH grant. The North-East reconstruction funds is a funding line that is provided by the federal government and some international donor agencies for the rehabilitation of the affected areas in the North-Eastern zone and encourage resettlement of IDPs post Boko Haram crisis. It is hoped that the three funding sources will be addressed with the advocacy to State-level government and non-government structures in all three States during the lifetime of the TB REACH project.

Table 2: Long-term Funding Plan

| Organisation | Funding plan |
| --- | --- |
| NTP | Low case finding a serious concern. So, if project proved successful there is a possibility of uptake of project activities. |
| SACA | Domestic funding will be included into annual budget and can be achieved through advocacy to the agencies before annual budget preparation. |
| North-East  Reconstruction Funds | Advocacy to the agencies SMOH |

(This box will be visible by the grantee)

| With the Boko Haram insurgency still unabated, the creation of emergency camps for IDPs and the need for organised funding of TB case finding in IDP camps will continue beyond the TB REACH project in these 3 States. The chance of inclusion of active case finding interventions, if the TB REACH project is successful with its advocacy plans is high. |
| --- |

1. Partnerships

According to the grantee, table 3 shows the range of existing partners and the expected relationships with the TB REACH project. Besides the NTP and the host communities where the IDP camps are, three other organisations are mentioned, including the WHO, the State PHC Development Agency and State Emergency Management Agency.

Table 3: Existing Relevant Partnerships

| Organisation | Quality of relationship |
| --- | --- |
| NTP | Already engaged right from time of proposal development, staff will participate in project monitoring, attend zonal and annual review meeting  State programme officers will be participating in supervision and monitoring |
| Other relevant stakeholders: such as SPHCDA, SEMA | Advocacy is planned to engage relevant stakeholders |
| Community | advocacy to the gate keepers during implantation |

(This box will be visible by the grantee)

| The project has a good network of national and international partnerships. The grantee believes the quality of the relationship the grantee currently has with these partners may benefit the sustainability of the project |
| --- |

1. Organisational Capacity

The grantee has an established structure for conducting advocacy for HIV/AIDS control over the years. This structure has identified and engaged key stakeholders in the States including community leaders, religious leaders, top Government officials and Civil Society Organizations as well as the media. This TB Reach project will leverage on this structure. The agencies for AIDS control in the 3 States is Chaired by the Governors in each of the States; this also provides an opportunity to mobilize more resources for sustaining this intervention. Other strategies planned include:

- Presence of functional vehicles which can be used for advocacy
- Working through states structure to make our advocacies more effective
- Presence of advocacy team in SACAs

(This box will be visible by the grantee)

| Gombe SACA is an agency for HIV/AIDS control in one of the three States to be involved in this TB REACH project. It will require strong functional and effective partnerships with the State TB/Leprosy Programmes of the all 3 States to be successful with the implementation of its activities. The claim of existing advocacy structures will be vital to the success and sustainability of the TB REACH interventions and adds to the proof of the sufficiency of the agency’s organisational capacity to deliver the project. |
| --- |

1. Communications

According to the response of the grantee, there is a plan to develop a policy brief for the dissemination of the results of TB REACH with support from McGill University. The current plan is to disseminate information about the project to the national and state governments. It also includes dissemination of project activities through websites, writing articles for publication and presentation at conferences, and presenting updates of the project in annual NTP review meeting.

(This box will be visible by the grantee)

| In the grant proposal, there is also the plan to share the project results with the community-level stakeholders in the advocacy teams, who will in turn use the information to solicit the support of government authorities. |
| --- |

**Advocacy Expected Results**

The grantee’s expectations from the advocacy to the various organisations at regional, and State levels are listed in table 4. These are in line with the results outlined in the grant proposal. Specifically, the grantee expects that the funding of the intervention activities post TB REACH will be included in the budget of the State TB control programmes of the three States to be covered by the project. Table 4 shows the grantee also expects that State AIDS Control Agencies (SACA) in the three States to support the provision HIV test kits and logistics for supervision of the project. In addition, there is the expectation that the Northeast restoration fund will provide fund to support the scale up of the project to a wider area after the current TB REACH grant stops. In the proposal, there are also the expectations that through the advocacy the intervention activities will become integrated into existing government structures and new partnerships with other government agencies.

Table 4: Expected Results of Advocacy

| Organisations | Expected result |
| --- | --- |
| NTP | Inclusion in future support to the areas |
| State programmes | Inclusion in annual budget in the 3 states |
| Northeast reconstruction funds | Funds allocated to the intervention for scaling up |
| Gombe SACA | Funds for procurement of Test kits, vehicles for supervision |
| SACA | Provision of test kits, vehicles for supervision |

(This box will be visible by the grantee)

| These expectations are in line with the sustainability plan in the grant proposal. At the moment, besides the availability of budget lines for TB control in the 3 States, the Governments (State and LGA) are not contributing to the case finding components. The North-East reconstruction fund support may not be adequate to fund the project after TB REACH grant stops. While GomSACA and the 2 other SACAs intensify advocacy towards stimulating these funding opportunities to the project, the most feasible expectation will be support of the community screening outreaches using HCT by SACA, since mobilising communities for HIV testing and counselling is already one of the core functions expected of the HIV/AIDS control programme in the States. Furthermore, results from this intervention would be integrated into the advocacy kit which will likely stimulate more funding for TB Control in the State. |
| --- |

# Populations

Table 5 shows the population figures reported by the grantee. The evaluation and control population are for 2016 projected from the 2006 National Census using 2.8% growth rate. The target IDP population given for the BV report has been adjusted from those from the IOM assessment figures reported in the proposal.

Table 5: Size of the populations reported by grantee after Grantee meeting in Bangkok:

| Population | Adamawa | Gombe | Yobe | Total Size | % |
| --- | --- | --- | --- | --- | --- |
| LGAs | 4 | 4 | 4 | 12 | - |
| Evaluation Pop | 960,960 | 1,446,146 | 955,239 | 3,362,345 | 100% |
| Target Pop (IOM figures 2016) | 159,445 | 69,444 | 112,671 | 341,761 | 10.2% |
| Target Pop  (Grantee’s edited figures) | 143,642 | 62,440 | 101,503 | 307,585 | 9.20% |
| Control Pop | 821,339 | 689,638 | 950,397 | 2,461,373 | 73.2% |
| EP : CP | 1.17 | 2.10 | 1.01 | 1.37 | - |
| TP : EP (IOM) | 0.16 | 0.04 | 0.12 | 0.10 |  |
| TP : EP (Grantee) | 0.0952 | 0.0275 | 0.0677 | 0.0583 | 0.0952 |

Target Population

According to IOM figures reported in the grant proposal, there are a total of 341,761 IDPs living in the 12 LGAs in the entire project area, with distribution of 46.7% (159,445) in Adamawa, 33.0% (159,445) in Yobe and 20.3% (69,444) in Gombe. Thus the grantee adjusted the size of the target population from 341761 to 307,585 (Table 5). The current size means the target population is smaller than the evaluation population that will be used to calculate additional notifications, being barely 9%, indicating a risk of dilution of the impact of the project. 90% of the total IDP population in the 3 States were targeted for the intervention in 4 key LGAs housing the IDPs in each States. The high risk of TB among the IDPs would be a factor that could mitigate against the anticipated dilution. Furthermore, TB case notification in these 3 States are among the lowest in the country. Therefore, the ACF activity will mop up the existing unidentified TB cases among the host community.

The ratio of IDPs based on the IOM report guided the distribution of the adjusted targets.

Evaluation and Control Populations

Table 5 shows that by comparison, the size of the control population is at least 73% the size of the evaluation population, with an EP:CP ratio that is within the required range of 0.33-3. The population sizes disaggregated by the component States, gives State EP:CP ratios that are also within this range. So the evaluation and control population sizes are therefore within a comparable range.

# Evaluation population

Table 6 shows the TB notifications in the evaluation population in 2016 by the NTP broken down by its component LGAs. A total of 3354 of all forms of TB cases were notified in 11 of the 12 LGAs, and 47.7% were Sputum bacteriological positive. No data is reported for Gujba LGA for the entire 12 quarters from 2014 to 2016. This was because the Boko Harram insurgency sacked the entire LGA population. This project will therefore be an opportunity to launch a TB programme in Gujba LGA. Calculating the rates for the 11 LGAs with TB data (excluding the population of Gjuba LGA in Yobe State), the corrected notification rates per 100,000 would be 105.11 and 50.11 for all forms and bacteriologically positive patients respectively.

Figure 1 shows the 2016 TB notifications of all forms which ranged from 65 patients in Fune LGA in Yobe State to 922 in Gombe LGA in Gombe State, indicating a 14 times difference between the LGA with the lowest and highest TB notifications in the evaluation population. However, figure 1 shows the notification in Gombe is outstanding, seeing it is about 2 times the second highest notification of 448 in Yola South in Adamawa. Figure 2 shows the range of standardised notification rates per 100,000 population from 16 in Fune LGA to 288 in Damaturu LGA in Yobe State. Still Gombe has the highest Bacteriologically positive notiifications of 101.6 per 100,000 population.

Table 6. TB Notifications in the Evaluation Population 2016 (disaggregated by LGAs)

| State | LGAs (BMUs) | Population | Notifications: New and Relapse B+ (N+R) | Notifications all forms (AF) | Rate* B+  (N+R) | Rate * AF | Bac+ (N+R) as % of AF |
| --- | --- | --- | --- | --- | --- | --- | --- |
| Adamawa | Yola north | 261,299 | 211 | 376 | 80.75 | 143.90 | 56.1% |
|  | Yola south | 256,501 | 166 | 448 | 64.72 | 174.66 | 37.1% |
|  | Mubi South | 169,945 | 94 | 214 | 55.31 | 125.92 | 43.9% |
|  | Fufore | 273,214 | 168 | 188 | 61.49 | 68.81 | 89.4% |
|  | Total | 960,960 | 639 | 1,226 | 66.50 | 127.58 | 52.12% |
| Gombe | Akko | 445,306 | 66 | 148 | 14.82 | 33.24 | 44.6% |
|  | Funakaye | 311,174 | 109 | 132 | 35.03 | 42.42 | 82.6% |
|  | Gombe | 353,237 | 359 | 922 | 101.63 | 261.01 | 38.9% |
|  | Y/Deba | 336,429 | 105 | 161 | 31.21 | 47.86 | 65.2% |
|  | Total | 1,446,146 | 639 | 1,363 | 44.19 | 94.25 | 46.88% |
| Yobe | Damaturu | 116,007 | 101 | 334 | 87.06 | 287.91 | 30.2% |
|  | Fune | 396,416 | 42 | 65 | 10.59 | 16.40 | 65.5% |
|  | Gujba | 171,462 | 0 | 0 | 0.00 | 0.00 | - |
|  | Potiskum | 271,354 | 178 | 366 | 65.60 | 134.88 | 48.6% |
|  | Total | 955,239 | 321 | 765 | 33.60 | 80.08 | 41.96% |
| EP Total | | 3,362,345 | 1,599 | 3,354 | 47.56 | 99.75 | 47.7% |

*Rate = notifications/100,000 population

The percentage of bacteriological positive among all forms notifications in 2016 ranged is lowest in Damaturu LGA in Yobe State and highest – more than 80% in Funakaye in Gombe State and Fufore in Adamawa (Figure 3). The other three of the four LGAs with % outside the validity range of 30% - 60%, include Y/Deba, Fune and Funakaye. The overall percentage is approx. 48% in the evaluation population with six LGAs having % above this overall figure. Remarkably the three LGAs reporting the higher absolute notifications or notification rates:Damaturu, Yola South and Gombe, also detected the lower percentages of Bacteriological positive patients, below the overall % for the evaluation population. The overall 48% is within the validity range of 30-60%, but lower than the national figure of 68% (2015 data) reported by WHO^[[2]](#footnote-2)^ by up to 20% (more than the 15% maximum difference required). According to the grantee’s explanation, the high smear positivity rates of new TB patients observed in the 4 LGAs was due to poor access to MOs for the clinical diagnosis of TB in these LGAs, thus Health Workers may register transfer-in TB cases, referred cases TB cases and B+ TB cases on treatment. Also contributory is the increase in access to GeneXpert machine especially among HIV clients. Thus more B+ cases are diagnosed and registered by HWs in these LGAs

# Control population

Table 7 shows the TB notifications in the control population (12 LGAs in the same 3 States: 4 in each State as the LGA distribution in the evaluation population). There are generally lower TB notifications of both categories of bacteriologically positive new + relapse patients and all forms in the control population than in the evaluation population (Table 8).

Table 7: TB notifications in the control propualtion in 2016

| State | LGA | Population | Notification B+ (N+R) | Notifications (AF) | Rate* B+ (N+R) | Rate* AF | B+ (N+R) as % of AF |
| --- | --- | --- | --- | --- | --- | --- | --- |
| Adamawa | Lamurde | 148,680 | 44 | 61 | 29.59 | 41.03 | 72.1% |
|  | Jada | 222,055 | 93 | 102 | 41.88 | 45.93 | 91.2% |
|  | Ganye | 216,275 | 125 | 150 | 57.80 | 69.36 | 83.3% |
|  | Guyuk | 234,329 | 50 | 56 | 21.34 | 23.90 | 89.3% |
|  | Total | 821,339 | 312 | 369 | 37.99 | 44.93 | 84.6% |
| Gombe | Gidam | 165,838 | 12 | 12 | 7.24 | 7.24 | 100.0% |
|  | NGURU | 198,540 | 91 | 227 | 45.83 | 114.33 | 40.1% |
|  | Karasuwa | 141,021 | 18 | 18 | 12.76 | 12.76 | 100.0% |
|  | Bade | 184,239 | 78 | 89 | 42.34 | 48.31 | 87.6% |
|  | Total | 689,638 | 199 | 346 | 28.86 | 50.17 | 57.5% |
| Yobe | Dukku | 273,086 | 28 | 42 | 10.25 | 15.38 | 66.7% |
|  | Balanga | 280,150 | 80 | 98 | 28.56 | 34.98 | 81.6% |
|  | Katungo | 197,450 | 51 | 213 | 25.83 | 107.88 | 23.9% |
|  | Shongom | 199,711 | 40 | 55 | 20.03 | 27.54 | 72.7% |
|  | Total | 950,397 | 199 | 408 | 20.94 | 42.93 | 48.8% |
| CP Total | | 2,461,374 | 710 | 1123 | 28.85 | 45.62 | 63.2% |

*Rate =notifications/100.000 population

Table 8: Comparison of 2016 total notifications in evaluation and control populations

| State | # LGA | Population | Notification B+ (N+R) | Notifications (AF) | Rate* B+ (N+R) | Rate* AF | B+ (N+R) as % of AF |
| --- | --- | --- | --- | --- | --- | --- | --- |
| National^ | 740 | 182,000,000 | 61,597 | 90584 | 33.84 | 49.77 | 68% |
| EP Total | 12 | 3,362,345 | 1,599 | 3,354 | 47.56 | 99.75 | 48% |
| CP Total | 12 | 2,461,374 | 710 | 1,123 | 28.85 | 45.62 | 63.2% |
| Difference | 0 | 900971.43 | 889 | 2231 | 18.71 | 54.13 | -15.5% |

^WHO available figures for 2015. 2016 figures unknown.

The differences in Table 8 show that both B+ (N+R) notifications and the AF notifications are lower in the control population than in the evaluation one. As a result, the two notifications rates in the evaluation population are about twice higher than the rates in the control population. Nevertheless, the B+ (N+R) as % of AF is higher in the control population than in the evaluation population, with a negative EP-CP difference of 15.5%, but different from the national % by a mere 4.8%, which is much less than the 15% maximum difference. So, there are marked differences in absolute number and rates of notifications between the EP and CP. The reason why the %B+ is particularly low in the EP still should be determined. Are these differences illustrated in figure 4 statistically significant to make the two populations incomparable for evaluation of the impact of the TB REACH? I do not know. But it may be easier to demonstrate the impact of project on case notification rates in low LGAs than in high. The percentage of Tb notifications in the IDP camps not known. But it is possible that the poorer access to TB diagnosis in IDP camps and host communities may be responsible.^[[3]](#footnote-3)^ Figure 4 shows this difference in B+ (N+R) as % of AF between the EP and CP is more marked in Adamawa State, where the control figure reached as high as 85%, and the EC-CP difference is 32.5%.

However, Table 7 shows that the figures of B+ (N+R) as % of AF was higher than the considered validity range of 30%-60% in 9 of the 12 LGAs in the control population, compared to just 4 in the evaluation population. The same explanation of poor access to medical diagnosis still holds here.

# Notifications in evaluation and control population over time

Overall Quarterly Notification Trends

The quarterly notifications in the evaluation and control populations over the 12 quarters from 2014 to 2016 are shown in tables 9 and 10 respectively. In the evaluation population, table 9 shows that a total of 9990 of notifications of AF were reported, with 4571 (45.8%) being bacteriologically confirmed PTB patients. The lowest notifications were in the fourth quarter of 2014, and the highest were in the first quarter of 2016. Table 10 shows that the total notifications reported for the 12 quarters in the control population was almost 3 times lower that those notified in the evaluation population, and the bacteriologically confirmed N&R PTB notifications in the CP were also more than two times (2.2 x) lower than in the EP.

Figure 5 compares the quarterly trends of AF notifications in the two populations. While the trend lines show the notifications in the control population were generally more stable than in the evaluation population, there are common depressions in notifications in the 4 quarters of 2014, and the first 3 quarters of 2016 that occurred during the 12 quarters in both populations. The same quarterly trends are still visible in the quarterly notifications of Bac+ (N&R) PTB patients shown in figure 6 in both populations. However the trend lines reveal the overall rise in the notification of of bacteriologically confirmed new and relapse PTB from 4^th^ quarter of 2014 to 4^th^ of 2016 in both populations, but with a higher gradient in the evaluation population than in the control. The trends in both populations of quarterly percentage of Bac+ N&R PTB patients among all forms in figure 7 reveal there was a general depression in the effectiveness of active PTB case detection between the middle of 2014 and early 2016, before a marked rise to the highest peak by the end of 2016.

Table 9: Quarterly TB Notifications in EP during 12 quarters from 2014 to 2016

| Quarters | Pulmonary TB (PTB) | | | | | | Extrapulmonary TB (EPTB) | | | Total |
| --- | --- | --- | --- | --- | --- | --- | --- | --- | --- | --- |
|  | Bac+ confirmed | | | Clinically diagnosed | | | Bac+ or clinical | | |  |
|  | N+R | Other | Total | N+R | Other | Total | N+R | Other | Total | AF |
| 2014/1 | 403 | 4 | 407 | 439 | 20 | 459 | 91 | 1 | 92 | 958 |
| 2014/2 | 359 | 11 | 370 | 379 | 17 | 396 | 66 | 41 | 107 | 873 |
| 2014/3 | 341 | 6 | 347 | 246 | 9 | 255 | 42 | 34 | 76 | 678 |
| 2014/4 | 280 | 10 | 290 | 267 | 15 | 282 | 60 | 2 | 62 | 634 |
| 2015/1 | 319 | 6 | 325 | 341 | 4 | 345 | 49 | 39 | 88 | 758 |
| 2015/2 | 424 | 3 | 427 | 437 | 2 | 439 | 40 | 51 | 91 | 957 |
| 2015/3 | 391 | 9 | 400 | 414 | 4 | 418 | 60 | 45 | 105 | 923 |
| 2015/4 | 370 | 15 | 385 | 360 | 3 | 363 | 54 | 53 | 107 | 855 |
| 2016/1 | 431 | 6 | 437 | 425 | 2 | 427 | 70 | 32 | 102 | 966 |
| 2016/2 | 345 | 3 | 348 | 364 | 2 | 366 | 36 | 28 | 64 | 778 |
| 2016/3 | 364 | 7 | 371 | 271 | 2 | 273 | 54 | 24 | 78 | 722 |
| 2016/4 | 459 | 5 | 464 | 340 | 2 | 342 | 61 | 21 | 82 | 888 |
| Total | 4486 | 85 | 4571 | 4283 | 82 | 4365 | 683 | 371 | 1054 | 9990 |

Grantee’s response

The depression observed in 2014 was due to the rise in the magnitude of attacks from the insurgents which peaked in the 4^th^ quarter especially in Adamawa and Yobe States; 7 LGAs were over-ran by the insurgents in Adamawa State resulting to a shutdown on DOTS service delivery points in these LGAs. The depression in the second and third quarters of 2016 coincided with the period when there was shortage of recording tools (mainly the TB patient record cards) and anti-TB drugs which affected the enrolment of TB patients on treatment.

Table 10: Quarterly TB Notifications in CP during 12 quarters from 2014 to 2016

| Quarter | Pulmonary TB (PTB) | | | | | | Extrapulmonary TB (EPTB) | | | Total |
| --- | --- | --- | --- | --- | --- | --- | --- | --- | --- | --- |
|  | Bac+ confirmed | | | Clinically diagnosed | | | Bac+ or clinical | | |  |
|  | N+R | Other | Total | N+R | Other | Total | N+R | Other | Total | AF |
| 14/1 | 200 | 5 | 205 | 94 | 5 | 99 | 14 | 2 | 16 | 320 |
| 14/2 | 162 | 5 | 167 | 109 | 2 | 111 | 22 | 0 | 22 | 300 |
| 14/3 | 157 | 1 | 158 | 60 | 4 | 64 | 10 | 8 | 18 | 240 |
| 14/4 | 139 | 1 | 140 | 59 | 12 | 71 | 5 | 0 | 5 | 216 |
| 15/1 | 163 | 1 | 164 | 106 | 1 | 107 | 9 | 8 | 17 | 288 |
| 15/2 | 177 | 2 | 179 | 94 | 11 | 105 | 3 | 2 | 5 | 289 |
| 15/3 | 183 | 2 | 185 | 96 | 10 | 106 | 14 | 3 | 17 | 308 |
| 15/4 | 174 | 4 | 178 | 91 | 6 | 97 | 7 | 7 | 14 | 289 |
| 16/1 | 172 | 4 | 176 | 132 | 1 | 133 | 9 | 7 | 16 | 325 |
| 16/2 | 168 | 2 | 170 | 90 | 4 | 94 | 9 | 2 | 11 | 275 |
| 16/3 | 163 | 1 | 164 | 52 | 1 | 53 | 11 | 0 | 11 | 228 |
| 16/4 | 207 | 2 | 209 | 71 | 8 | 79 | 4 | 3 | 7 | 295 |
| Total | 2065 | 30 | 2095 | 1054 | 65 | 1119 | 117 | 42 | 159 | 3373 |

Table 11: Quarterly notifications of AF in the LGAs of the Evaluation Population: 2014-2016

| LGAs | 2014 | | | | 2015 | | | | 2016 | | | | Total |
| --- | --- | --- | --- | --- | --- | --- | --- | --- | --- | --- | --- | --- | --- |
|  | Q1 | Q2 | Q3 | Q4 | Q1 | Q2 | Q3 | Q4 | Q1 | Q2 | Q3 | Q4 |  |
| Yola north | 84 | 71 | 76 | 72 | 93 | 93 | 90 | 73 | 95 | 73 | 105 | 103 | 1028 |
| Yola south | 101 | 92 | 75 | 47 | 109 | 153 | 105 | 108 | 138 | 101 | 88 | 121 | 1238 |
| Mubi South | 92 | 107 | 41 | 15 | 31 | 65 | 90 | 70 | 63 | 80 | 32 | 39 | 725 |
| Fufore | 66 | 61 | 38 | 53 | 38 | 37 | 46 | 47 | 51 | 50 | 31 | 56 | 574 |
| Akko | 46 | 37 | 45 | 36 | 39 | 75 | 54 | 40 | 63 | 31 | 22 | 32 | 520 |
| Funakaye | 39 | 40 | 24 | 22 | 35 | 39 | 40 | 34 | 30 | 36 | 24 | 42 | 405 |
| Gombe | 264 | 228 | 191 | 212 | 222 | 276 | 269 | 257 | 297 | 223 | 195 | 207 | 2841 |
| Yamaltu Deba | 40 | 34 | 49 | 40 | 44 | 72 | 50 | 47 | 46 | 37 | 30 | 48 | 537 |
| Damaturu | 82 | 84 | 70 | 51 | 39 | 66 | 62 | 80 | 71 | 71 | 86 | 106 | 868 |
| Fune | 18 | 21 | 10 | 7 | 15 | 10 | 15 | 8 | 16 | 10 | 20 | 19 | 169 |
| Gujba | 0 | 0 | 0 | 0 | 0 | 0 | 0 | 0 | 0 | 0 | 0 | 0 | 0 |
| Potiskum | 126 | 98 | 59 | 79 | 93 | 71 | 102 | 91 | 96 | 66 | 89 | 115 | 1085 |
| Total | 958 | 873 | 678 | 634 | 758 | 957 | 923 | 855 | 966 | 778 | 722 | 888 | 9990 |

Disaggregation by LGAs

Tables 11 and 12 show the quarterly notifications of AF disaggregated by LGAs in the evaluation and control populations respectively during the 12 quarters reviewed. Figures 8 and 9 illustrate the different LGA quarterly trends. Figure 8 shows in the Evaluation that apart from the outstanding trend in Gombe LGA, the highest peaks of quarterly AF notifications in the other LGA were either about 150 or less all through the period. The trend in Gombe LGA increased from Q3 2014 to its highest peak in Q1 2016, with observable depressions during the quarters of 2014 and 2016. The same trends are observed in a few LGAs such as Yola South, Mubi South and Akko. Figure 9 shows that less distinction of trends is seen in the LGAs in the control population.

Table 12: Quarterly notifications of AF in the LGAs of the Control Population: 2014-2016

| LGA | 2014 | | | | 2015 | | | | 2016 | | | | Total |
| --- | --- | --- | --- | --- | --- | --- | --- | --- | --- | --- | --- | --- | --- |
|  | Q1 | Q2 | Q3 | Q4 | Q1 | Q2 | Q3 | Q4 | Q1 | Q2 | Q3 | Q4 |  |
| Lamurde | 22 | 25 | 18 | 18 | 20 | 12 | 13 | 14 | 15 | 16 | 13 | 17 | 203 |
| Jada | 34 | 20 | 27 | 29 | 25 | 27 | 24 | 26 | 27 | 27 | 20 | 28 | 314 |
| Ganye | 41 | 31 | 26 | 29 | 40 | 24 | 41 | 41 | 35 | 35 | 39 | 41 | 423 |
| Guyuk | 26 | 21 | 20 | 10 | 12 | 11 | 17 | 14 | 13 | 15 | 11 | 17 | 187 |
| Balanga | 31 | 21 | 25 | 11 | 27 | 26 | 24 | 32 | 31 | 29 | 18 | 20 | 295 |
| Dukku | 4 | 10 | 19 | 9 | 12 | 13 | 19 | 9 | 14 | 7 | 7 | 14 | 137 |
| Kaltungo | 50 | 38 | 24 | 34 | 51 | 60 | 59 | 52 | 77 | 53 | 34 | 49 | 581 |
| Shongom | 5 | 5 | 7 | 10 | 9 | 12 | 15 | 10 | 21 | 12 | 4 | 18 | 128 |
| Bade | 14 | 19 | 19 | 23 | 17 | 21 | 24 | 25 | 21 | 23 | 19 | 26 | 251 |
| Geidam | 5 | 10 | 3 | 0 | 1 | 2 | 9 | 2 | 3 | 3 | 2 | 4 | 44 |
| Karasuwa | 4 | 2 | 5 | 2 | 6 | 4 | 4 | 3 | 2 | 5 | 7 | 4 | 48 |
| Nguru | 81 | 98 | 47 | 41 | 68 | 77 | 59 | 61 | 66 | 50 | 54 | 57 | 759 |
| Total | 317 | 300 | 240 | 216 | 288 | 289 | 308 | 289 | 325 | 275 | 228 | 295 | 3370 |

Similarly, figures 10 and 11 show that LGA trends of the Bac+ (N&R) PTB notifications in the evaluation and control population. In the evaluation population, apart from Gombe that shows a rising wavy trend during the 12 quarters, trends in the other LGAs are not distinguishable. Similarly, in the control population in figure 11, only Ganye LGA shows a rising trend distinguishable from the others.

Target additional notifications

Table 13: Ratio of Target additional notifications to Baseline Annual Notifications EP

| Process indicators | Notifications Bac+ (N&R) | Notifications AF | % Bac+/Total AF | NR* Bac+ | NR* AF |
| --- | --- | --- | --- | --- | --- |
| House to House Screening | 2,239 | 2,895 | 77% |  |  |
| HCT Community Outreach Screening | 470 | 627 | 75% |  |  |
| Targeted total (TP) | 2,709 | 3,522 | 77% | 80.57 | 104.75 |
| Baseline (2016 annual) EP Total | 1,599 | 3,354 | 48% | 47.56 | 99.75 |
| Baseline (2016 annual) CP Total | 710 | 1,123 | 63% | 28.85 | 45.62 |
| Nigeria (2015)^[[4]](#footnote-4)^ | 61,597 | 90,584 | 68% | 33.84 | 49.77 |
| Expected Additionality  (TP-EP difference) | 1,110 | 168 | 29% | 33.01 | 5.00 |
| Ratio of Target additional/Baseline Annual notification (EP) | 0.69 | 0.05 |  |  |  |

From the spreadsheet of process indicators updated, the target additional notifications of the TB REACH project are shown in table 13. The ratio of target annual Bac+ notifications to annual baseline notifications is higher than the required minimum of 0.2

Besides, the target 77% of Bac+ among the AF detected from the two interventions is outside the validity range of 30%-60%. As the active case finding effort will be applied on a target population of IDPs that are less than 10% of the evaluation population, there is a high risk of dilution of the impact or results attributable to the implementation of this TB REACH project.

Grantee’s response

Although the target of 77% is outside the validity at this point, it should be noted that with more territorial gains by the military in the war against the insurgents and the continuous release of captured hostages, there could be an increase in the target population. Further more, according to the risk assessment conducted by KNCV in Nigeria, the risk of TB among IDPs is the highest in comparison to other key populations.

# Activities

Review of the Workplan

The grantee wants to implement the activities in a state-by-state phased implementation strategy and plans to begin in Adamawa State. According to the project workplan in Annex 2, the grantees plans to use the first quarter for preparation, and begin implementation of active case finding from the second quarter. Table 14 shows a synthesis of the workplan in annex 2. It reveals the project wants to execute 16 activities grouped into 4 broad headings. Half of them are preparatory activities to be completed by the end of June in the first quarter. The other three categories will start simultaneously from July in the second quarter. Obviously, according to the workplan, advocacy activities will be done only during the preparation stage and no dissemination activities are planned besides the operational research. No specific dates are given

Table 14: A synthetic reorganisation of the project workplan

| Preparation:  Take-off and stage setting | Implementation:  Active Case Finding | Progress Monitoring | Impact Evaluation and Dissemination |
| --- | --- | --- | --- |
| - Orientation of State Project Teams - Planning meetings - Mpping partners and stakeholders - Stakeholding meeting and advocacy visits - Procurement and logistics - Recruitment of CBOs and CVs - Training: CBOs, CVs - Refresher training of DOTs and Lab workers | - Screening and Referral of TB presumptive cases by CVs - Contact examination of TB index cases by CVs - Quarter outreach and awareness creation by CBOs* | - Monthly and quarterly suoervision of CBOs, CVs - Monthly Review meetings of CBOs, CVs - Attendance of the State quarterly review meetings - Quarterly progrss review meetings with State project teams | - Conduct Operational Research* |
| April to June 2017 | From July 2017 | From July 2017 | From 2017 |

*included in the M&E category

Implementers and workload

CBOs

- 4 CBOs – 1 per State of adamawa and Yobe while 2 in Gombe State ( CBO to mobilise communities in 12 LGAs for 3 quarterly outreaches and conduct HCT and TB Screening of community members and supervision of CVs. But the number of IDP camps and communities in each LGA is unknown. But all CBOs are expected to screen 58797 community members. This means an average of 3,266 people per CBO per outreach. How many staff will each CBO deploy to execute the outreach activities? This is unknown.

Grantee’s response

The number of IDP Camps has been fluctuating due to the dynamics in movement of the IDPs from within Nigeria and outside from the Cameroons. However, each of the 8 LGAs in Adamawa and Yobe has at least 1 large permanent IDP camp that holds thousands of IDPs at any given time in addition to several IDP host communities. Gombe State has no IDP Camps but has IDP host communities in all the 4 intervention LGAs.

Considering the overhead costs of engagement of CBOs, the project now plans to engage 4 CBOs instead of 6. As for the quarterly community outreach by the CBOs, the project will ensure that in addition to the 3 core staff of each CBO (Project Manager/Officer, Monitoring and evaluation officer and project accountant), each of the CBOs will draw at least 6 volunteers (2 per LGA) from the already trained CVs in each of the 4 LGAs, 4 HIV formally trained counsellors (1 per LGA) from the existing pool of counsellors in the SACAs and the existing 4 LGTBLS (1 per LGA) making a total of 7 persons per LGA per outreach.

CVs

- The number of CVs has been reviewed upward to 15 per LGA making a total of 60 per State and 180 for the 3 States to screen a target of 248,788 people. Therefore, each CV is expected to verbally screen 1,382 IDPs for symptoms of TB during the house-to-house intervention over 3 quarters.
- In addition, they will also screen 1,788 contacts of 447 index TB patients identified and referred by CBOs. With this target, each CV may screen about 3,170 persons. This implies that each CV will screen 264 persons per month The number of IDP camps and host communities are in each LGA is not known. Clearly this is a lot of work for each CV.

State Project Team

3 State Project Teams - 1 per State and Each State Project Team (SPT) will be made up of 5 persons (SPT Leader who is the head of SACA in each State, the State TB Programme Officer, the State Laboratory Quality Assurance Officer, Representative of the Primary Health Care Agency and the M & E Officer from the CBO in the State). The role of the SPT include:

1. The coordination of the intervention across the 4 LGAs in each State
2. Supervision of the CBOs, LGTBLS and Community Volunteers
3. Organising monitoring meetings with the Lab staff from Gx sites, CVs and LGTBLS
4. Developing monthly reports of activities to GomSACA
5. Addressing challenges that may occur in the course of project implementation

In each state, the TB focal person should monitor the process of verbal screening of TB presumptive persons, the GeneXpert testing, referral or linkage to DOT and following-up of treatment and outcomes with the LGA TBLS. The HIV focal person should monitor the process of HCT, and the referral of all HIV+ patients to ART and DOTs centres.

Gombe SACA

- Gombe SACA staff – They will execute the preparation activities and central organisation and coordination, and the day to day management and trouble-shooting of the project throughout its phased implementation period.
- GomSACA has identified 3 core members to form the GomSACA TB REACH Project Team. The are the Project Manager, the Monitoring and Evaluation Officer and the Project Accountant. Their role include:

1. The coordination of the intervention across the 3 States
2. Data collation from the 3 States and subsequent transmission to TB REACH
3. Linkage with the NTP and other key stakeholders at the National level
4. Ensure uninterrupted supply of commodities and tools required in the field for the intervention
5. Ensure that the Gx equipment in the intervention areas remain functional

Community Stakeholders Advocacy teams

- Although missing in the workplan, the advocacy and sustainability plan discussed earlier that teams of community stakeholders will be mobilised to make advocacy visits to key government agencies in each state, to solicit for political and budgetary support to sustain or scale up the intervention after TB REACH funding stops. The workplan states that the advocacy visits will be done in the first quarter. This amount and time of advocacy might not be enough. What will be the size of this team in each state and how will the membership be selected?

# Process indicators

Table 15 shows the Project Indicators (PI) according to the Pathway of TB Care (PoC) of the project, which will be tracked during the project term. Compared to the Bac+ and AF notifications of 1599 and 3354 in the evaluation population in 2016 (see tables 6 & 8), the target yields of B+ and AF from screening a total of 307,585 persons in the 3 States are shown in table 15. The different targets for numbers of presumptive TB persons and those of them tested shows the grantee has no aim to test everyone identified with TB symptoms after verbal screening. Table 15 shows that while 30,758 persons with TB symptoms are targeted, only 27,094 (88%) will be tested. Similarly not all confirmed TB patients will be registered for treatment: only 98% (6110/6231) are targeted for treatment. However the grantee gave assurance that all persons with presumptive TB will be tested and all diagnosed TB patients will be registered for treatment. A 96% treatment success rate is targeted.

Table 15: Process Indicators: Annual Targets

| Verbal screening of IDPs in Camps and host Communities | H2H IDP Screening | HCT Community Outreach Screening | Total |
| --- | --- | --- | --- |
| Numbers Needed to be Screened (NNS) | 248,788 | 58,797 | 307,585 |
| Number people with TB symptoms | 24,878 | 5,880 | 30,758 |
| Number people tested for TB | 22,390 | 4,704 | 27,094 |
| Number people confirmed TB B+ | 2,239 | 470 | 2,709 |
| Number people TB diagnosed AF | 2,895 | 627 | 3,522 |
| Number of people confirmed TB B+ registered for treatment | 2,230 | 447 | 2,677 |
| Number of people confirmed AF TB registered for treatment | 2,837 | 596 | 3,433 |
| Number people successfully completed treatment (B+) | 2,163 | 402 | 2,565 |
| Number people successfully completed treatment (AF) | 2,752 | 536 | 3,288 |
| Contact tracing Indicators: |  |  |  |
| - # of index patients |  | 447 |  |
| - # of contacts of index patients screened for TB |  | 1,788 (Average of 4 per index) |  |

House-to-House household screening:

1. Pre-screening steps – verbal screening for symptoms of TB e.g. cough of at least 2 weeks duration, using a pre-set chacklist
2. Work load per (community) health worker per day – according to proposed plan: On average, a CV is expected to visit at least 10 households in his or her designated area spending on average 30 minutes per household (household size of 4-6 members), the goal is to reach on average ~20 people per day or at a five-day work-week ~100 people per week. However this workload was estimated on the basis of the initial screening the total target population of 307, 585 by 180 CVs in 12 months in which each CV will screen about 3000 people by end of project.
3. Amount of undiagnosed TB in the community – Based on the national TB prevalence survey, the estimated prevalence of TB in the project area is about 17,618 of all forms, with about 10% in IDP camps. Actual AF case notifications by the end of 2016 it was 1,123. Thus, the number of undiagnosed TB is estimated as 16,495 in the 12 LGAs in the evaluation population. In the IDP camps and communities, the number undiagnosed being 10% is 1,650. Hence, the PoC in table 15 shows a targets detection of 1693 AF TB patients, just a a little above the estimated number undiagnosed TB.

HCT community outreach screening:

1. The proposed number of sessions and likely attendance

The proposed number of session is one per quarter per LGA i.e 12 in a Quarter and a total of 36 in 3 Quarters (project period). Each LGA is expected to reach at least 1000 people every quarter giving a total of 36,000 people in 12 LGAs through out the project period.

1. Presumptive TB rate – About 5880 are expected to have symptoms of TB in the community that will be screened using HCT community screening intervention.

# External Factors

The key external factors that might influence the project include:

- New insugency areas
- Raining reason
- Government funding inaccessible
- Closure of IDP camps
- Migration of IDPs in and out of the camps
- Strike by healthworkers for non-payment of salary

According to the grantee, Global funds and TB challenge are currently supporting active case finding in Slums in some selected States however none of the 3 states of our intervention is included, so we don’t expect any effect. Opening or closing of TB diagnostic and treatment centres is unlikely with on the period of project implementation as Global fund new project might take up to a year proposal wring has just commenced.

There is posibility of closing camps by Government and retunding of IDPs to their original communities which can be out side the project LGAs

A mitigation plan is needed.

# Data quality assessment

Data quality check will start from the monthly and quarterly supervisions by the CBOs, LGTBLS, State TB Programme and the SPT. This supervision will be done jointly with data validation. Data validation will also be conducted at the quarterly State TB Programme review meeting where data from the project will finally be segregated. These activities will be implemented with the aid of a checklist.

# Follow up for project patients on treatment

LGA supervision will be commisioned to follow up and report on the confirmed IDP patients put on treatment.

# Operational research

Has the grantee included operational research (OR) into the proposal?

Yes  No

If the grantee has included OR in the proposal, then answer the following:

Does the budget include a line for OR?

Yes  No

Does the proposal include an outline of the OR?

Yes  No

Is the outline of the proposal reasonable?

Yes  No

Would support of McGill be necessary in your opinion?

“Prevalence and treatment outcomes of TB, HIV and TB/HIV co-infection among IDPs in North-eastern Nigeria”

Protocol not yet developed

Already discussing with McGill on the OR

According to the proposal we are expected to start the OR from end of 1st Quarter, 2018

# Annexes

Annex 1: Maps of the 3 States showing the evaluation and control LG Areas


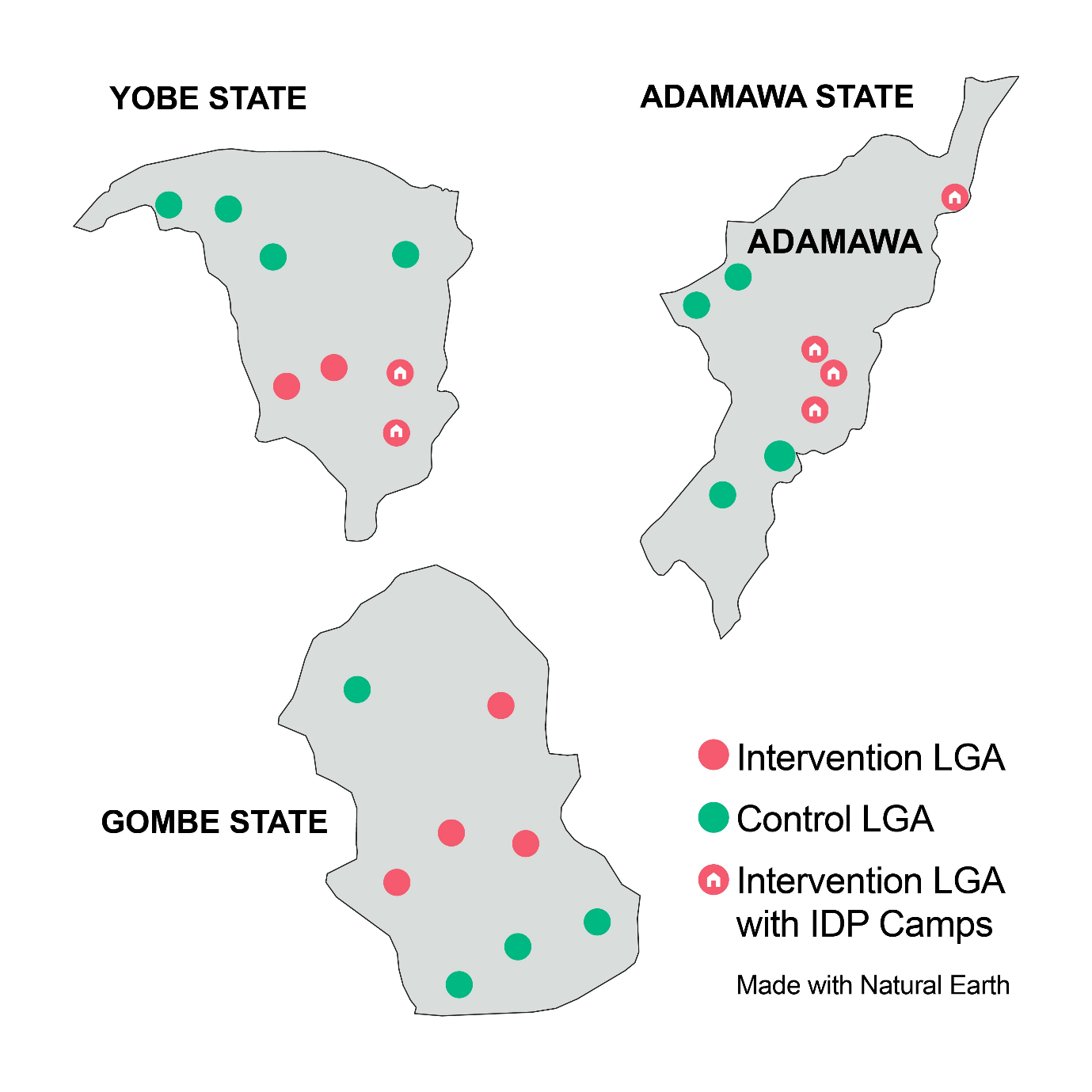


Annex 2: TB REACH Workplan

| **Objective** | **Activity** | **Implementing Individual(s) / Organization(s)** | **Timeline** |
| --- | --- | --- | --- |
| **Ensure proper project take up and setting the phase for project sustainability** | **1.1** Identification, formation and orientation of state project team in Adamawa, Gombe and Yobe. | GomSACA (4 members- TB focal persons, QA officer, HIV focal persons and M&E officer) | 1st to 2nd week of April |
|  | **1.2**. Core planning meetings | GomSACA/ State project teams | 3rd week of April |
|  | **1.3** Mapping of existing partners/stakeholders: | GomSACA/State Project Teams | 4th Week of April |
|  | **1.4**  Stakeholder meeting and Advocacy meetings/visit   - to relevant stakeholders in the 3 states - to health facilities (public, and private) within the IDPs camps and host communities | GomSACA/State Project Teams | 1st Week of May |
|  | **1.5** Procurement of items for field work like bags, IEC materials, data capturing tools, umbrellas, sputum boxes: | GomSACA | 1st to 3rd week of May (These will be used during the trainings) |
|  | **2,1** Identify and recruit Community Based Organization: | GomSACA/ State Project Teams | 1st to 2nd week of May |
|  | **2.2** Identify and recruit Community volunteers: | CBOs/State  Project Teams/Community Stakeholders | 2nd to 3rd week of May |
|  | **2.3** Training of community volunteers/CBOs (180 CVs and 6 CBOs): | GomSACA/State Project Teams | 1st to 2nd week of June |
|  | **2.4** Training/refresher training of DOTs and laboratory focal on TB/HIV and Genexpert screening: | GomSACA/State Project Teams | 3rd to 4th week of June |
| **Active case finding for TB and HIV among target population** | **3.1** Screening & referral for TB and HIV of target population to identify presumptive TB case and HIV+ by CVs: | CBOs/Community Volunteers | From 1st week of July, 2017 |
|  | **3.2** Conduct contact examination of all TB cases by CVs | CBOs/Community Volunteers | From 2nd week of July, 2017 |
| **Monitoring and Evaluation of the project** | **4.1** Conduct monthly supervision & monitoring of CBOs and CVs: | LGA TB Supervisor | From 3rd week of July, 2017 |
|  | **4.2** Quarterly outreach and awareness creation activities: | CBOs/Community Volunteers | From 1st week of August, 2017 |
|  | **4.3** Quarterly supervision of CVs & CBOs: | State TB Team | From 1st week of August, 2017 |
|  | **4.4** Quarterly supervision of CVs & CBOs: | State Project Team | From 1st week of August, 2017 |
|  | Quarterly supervision of CVs & CBOs: | GomSACA/Central Project Team | From 1st week of September, 2017 |
|  | Monthly review meetings for CBOs, CVs, LGA TB Supervisors, State TB Team and State Project Team: | GomSACA | From 1st week of July, 2017. |
|  | Participate in State Quarterly programme review meeting: | State Project Team | From end of 2nd Quarter |
|  | Quarterly review meeting by GomSACA for State Project Team and CBOs: | GomSACA | From end of 2nd Quarter |
|  | Conduct OR: | GomSACA | From end of 1st Quarter, 2018 |

1. Source of information: Grantee Application / proposal, 2015. [↑](#footnote-ref-1)
2. WHO. (2017). Nigeria Tuberculosis Profile. 2015 data. Retrieved from https://extranet.who.int/sree/Reports?op=Replet&name=%2FWHO_HQ_Reports%2FG2%2FPROD%2FEXT%2FTBCountryProfile&ISO2=NG&LAN=EN&outtype=html [↑](#footnote-ref-2)
3. Robert Stevens. Second Reviewer’s comments [↑](#footnote-ref-3)
4. WHO. (2017). Nigeria: TB profile. Online. Retrieved from: https://extranet.who.int/sree/Reports?op=Replet&name=%2FWHO_HQ_Reports%2FG2%2FPROD%2FEXT%2FTBCountryProfile&ISO2=NG&LAN=EN&outtype=html [↑](#footnote-ref-4)
