# Supplementary material for: Providing TB and HIV outreach services to internally displaced populations in Northeast Nigeria: Results of a controlled intervention study
Source: PLoS Med. 2020 Sep 9;17(9):e1003218. doi: 10.1371/journal.pmed.1003218 (PMC7480873; doi:10.1371/journal.pmed.1003218)
Supplement: S1 TREND Checklist — (DOCX) [file pmed.1003218.s002.docx]

| **Paper Section/**  **Topic** | **Item No** | **Descriptor** | **Reported?** | |
| --- | --- | --- | --- | --- |
|  |  |  | 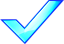 | **Pg #** |
| **Title and Abstract** | | | | |
| Title and Abstract | 1 | 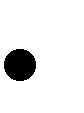 Information on how unit were allocated to interventions | 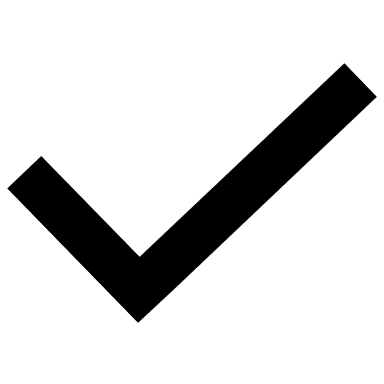 | Title, Abstract: 2^nd^ paragraph (methods and findings) |
|  |  | 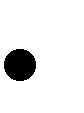 Structured abstract recommended | 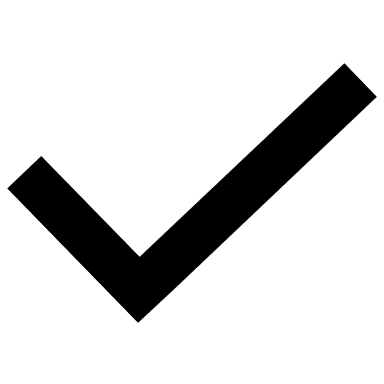 | Abstract is structured |
|  |  | 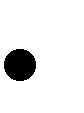 Information on target population or study sample | 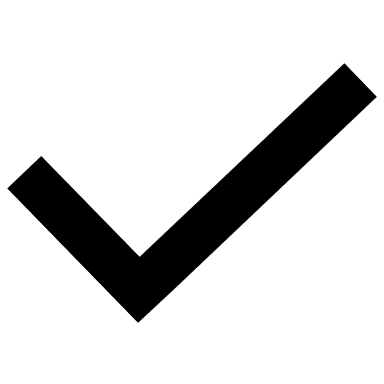 | Abstract contains information on target population and sample size |
| **Introduction** | | | | |
| Background | 2 | 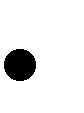 Scientific background and explanation of rationale | 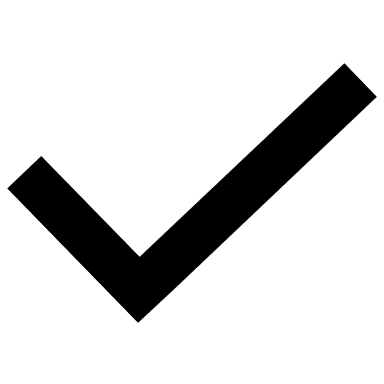 | **Introduction**, paragraphs 4-6 present background and rationale for intervention |
|  |  | 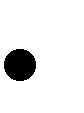 Theories used in designing behavioral interventions | 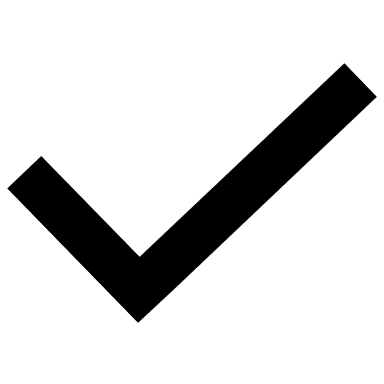 | **Methods** section presents methodology used for data assessment in paragraph 1. |
| **Methods** | | | | |
| Participants | 3 | 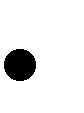 Eligibility criteria for participants, including criteria at different levels in recruitment/sampling plan (e.g., cities, clinics, subjects) | 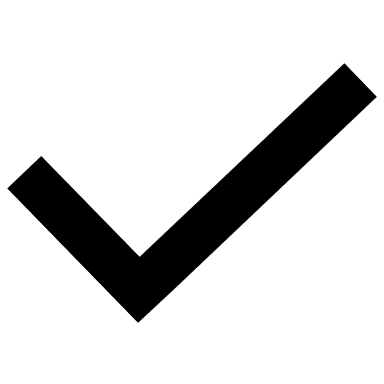 | All IDPs in areas mapped for the intervention were eligible for the intervention. The selection criteria of the intervention areas are described in **Methods** section paragraphs 3 (under *Intervention Area* heading) and paragraph 4 (under *Mapping IDP populations and*… heading) |
|  |  | Method of recruitment (e.g., referral, self-selection), including the sampling method if a systematic sampling plan was implemented | 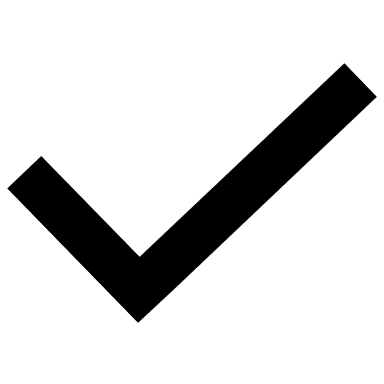 | The recruitment of intervention participants is described in paragraphs 13-17 of the **Methods** section under the heading of *Outreach Interventions for HIV and TB* |
|  |  | Recruitment setting | 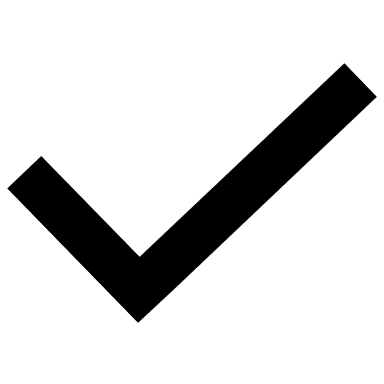 | The settings for intervention and recruitment are described throughout the introduction and can be also found in **Methods** paragraph 3 and 4 and paragraphs 13-17. |
|  |  | Settings and locations where the data were collected | 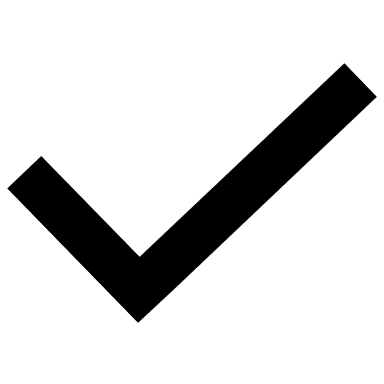 | Data collection settings are described in Methods paragraph 18 under the heading of *Result Retrieval and Linkage to Treatment* |
| Interventions | 4 | 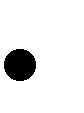 Details of the interventions intended for each study condition and how and when they were actually administered, specifically including: |  |  |
|  |  | o Content: what was given? | 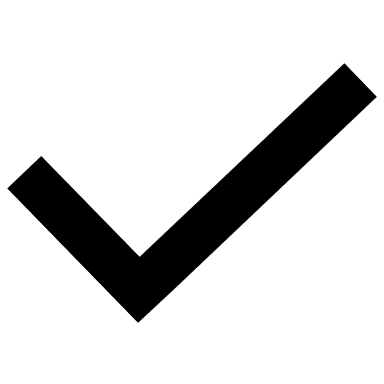 | Paragraphs 13-17 of the **Methods** section under the *Outreach interventions for HIV and TB* address the content of the intervention |
|  |  | o Delivery method: how was the content given? | 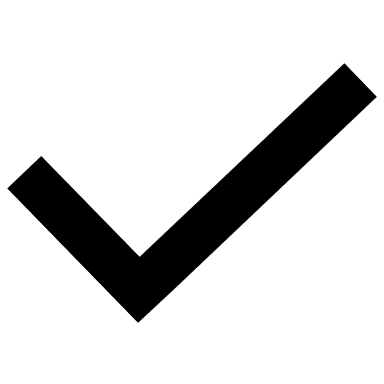 | Paragraphs 13-17 of the **Methods** section under the *Outreach interventions for HIV and TB* address how the content of the intervention was delivered |
|  |  | o Unit of delivery: how were the subjects grouped during delivery? | 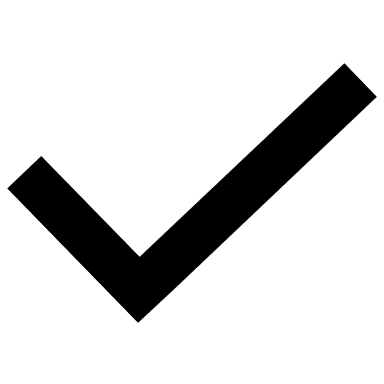 | Paragraphs 14-16 of the **Methods** section describe the groupings of different types of populations of IDPs for the intervention |
|  |  | o Deliverer: who delivered the intervention? | 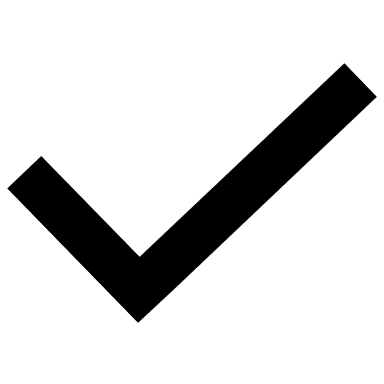 | Methods paragraph 15 sentence 4, paragraph 16 sentence 1, Methods paragraph 8 also identifies the workforce for the intervention |
|  |  | o Setting: where was the intervention delivered? | 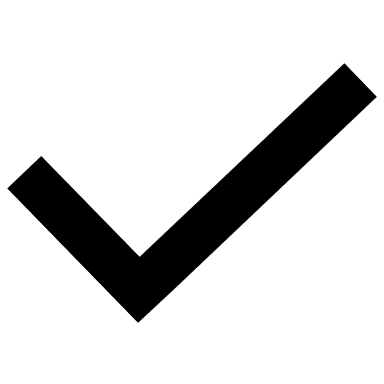 | The settings for the intervention are described throughout the **Introduction** section and can be also found in Methods paragraph 3 and 4 and paragraphs 13-17. |
|  |  | o Exposure quantity and duration: how many sessions or episodes or events were intended to be delivered? How long were they  intended to last? | 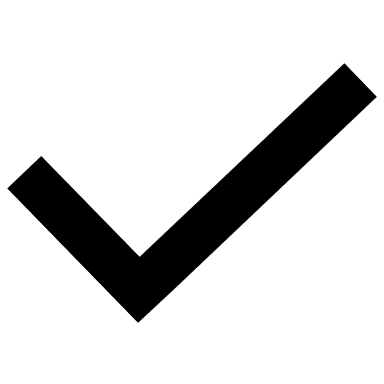 | Duration and frequency of interventions are described to the best of our ability in **Methods** paragraphs 13-18 |
|  |  | o Time span: how long was it intended to take to deliver the  intervention to each unit? | 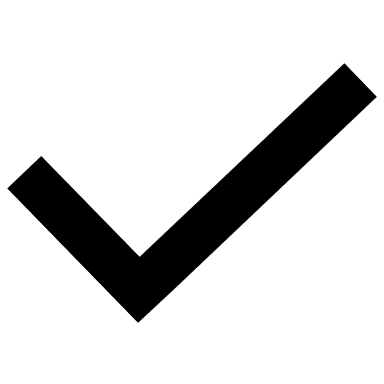 | The dates of the intervention are described in **Methods** section under *Intervention area* heading (paragraph 2 of Methods) |
|  |  | o Activities to increase compliance or adherence (e.g., incentives) | 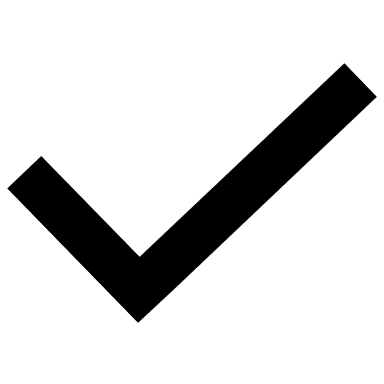 | Incentives are described in **Methods** paragraph 17 |
| Objectives | 5 | 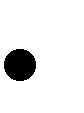 Specific objectives and hypotheses | 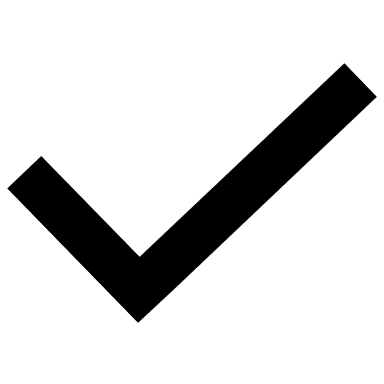 | We present the objective of the study in **Methods** paragraph 1. |
| Outcomes | 6 | 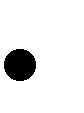 Clearly defined primary and secondary outcome measures | 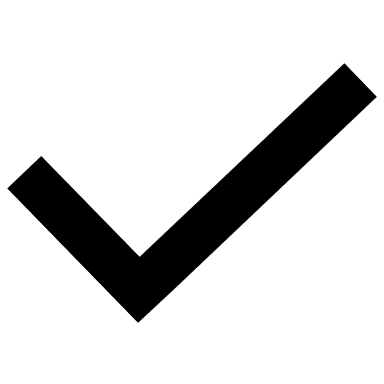 | **Methods** paragraph 19 sentences 3, 5, 6. |
|  |  | 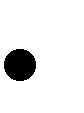 Methods used to collect data and any methods used to enhance the quality of measurements | 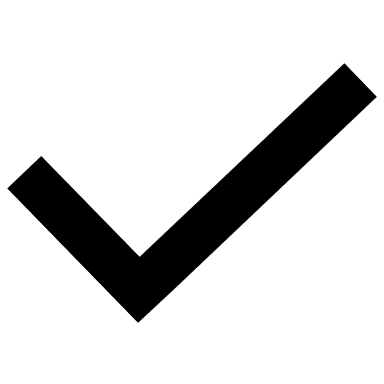 | **Methods** paragraphs 18 and 19 |
|  |  | 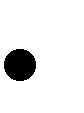 Information on validated instruments such as psychometric and biometric properties | 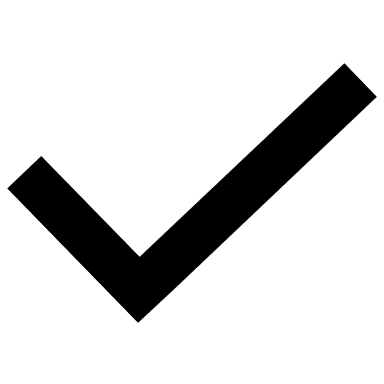 | **Methods** paragraph 19 |
| Sample Size | 7 | How sample size was determined and, when applicable, explanation of any interim analyses and stopping rules | 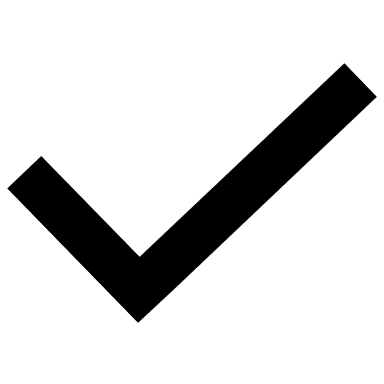 | **Methods** paragraphs 2-3 describe the selection of intervention and control populations and provide basis for this |
| Assignment Method | 8 | Unit of assignment (the unit being assigned to study condition, e.g., individual, group, community) | 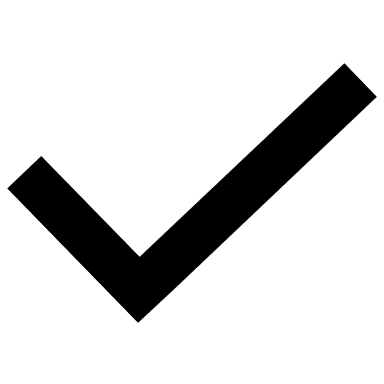 | **Methods** paragraphs 2-3 describe the selection of intervention and control populations and provide basis for this |
|  |  | Method used to assign units to study conditions, including details of any restriction (e.g., blocking, stratification, minimization) | N/A | Not applicable to our intervention |
|  |  | Inclusion of aspects employed to help minimize potential bias induced due to non-randomization (e.g., matching) | N/A | Not applicable to our intervention. |

| Blinding (masking) | 9 | 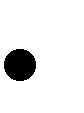 Whether or not participants, those administering the interventions, and those assessing the outcomes were blinded to study condition assignment; if so, statement regarding how the blinding was accomplished and how it was assessed. | N/A | Not applicable to our interventions |
| --- | --- | --- | --- | --- |
| Unit of Analysis | 10 | 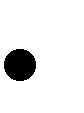 Description of the smallest unit that is being analyzed to assess intervention effects (e.g., individual, group, or community) | 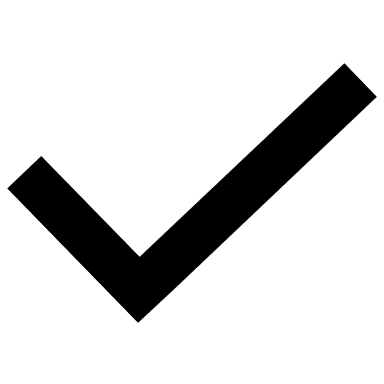 | **Methods** paragraph 19 under *Analysis* describes analysis |
|  |  | 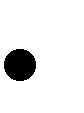 If the unit of analysis differs from the unit of assignment, the analytical method used to account for this (e.g., adjusting the standard error estimates by the design effect or using multilevel analysis) | 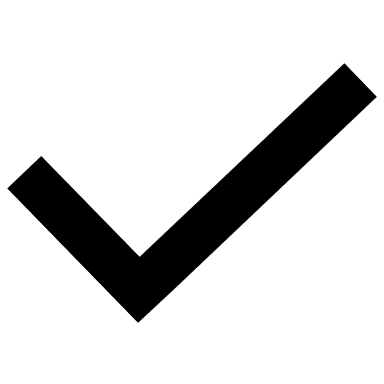 | **Methods** paragraph 19 under *Analysis* describes analytical methods used |
| Statistical Methods | 11 | 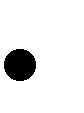 Statistical methods used to compare study groups for primary methods outcome(s), including complex methods of correlated data | 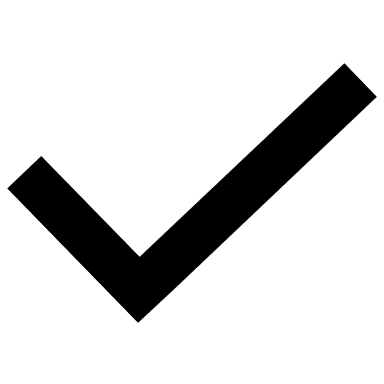 | **Methods** paragraph 19 under *Analysis* describes analytical methods used |
|  |  | 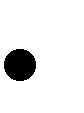 Statistical methods used for additional analyses, such as a subgroup analyses and adjusted analysis | 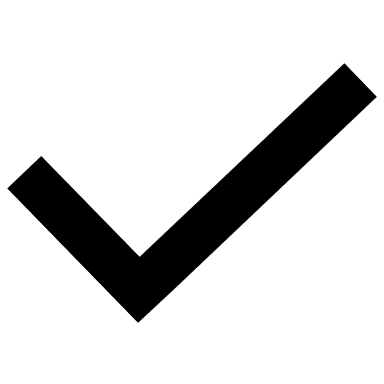 | **Methods** paragraph 19 under *Analysis* describes analytical methods used |
|  |  | Methods for imputing missing data, if used | N/A | N/A |
|  |  | Statistical software or programs used | 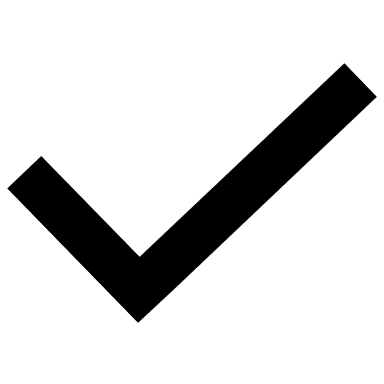 | **Methods** paragraph 19 under *Analysis* describes statistical software used |
| **Results** | | | | |
| Participant flow | 12 | Flow of participants through each stage of the study: enrollment, assignment, allocation, and intervention exposure, follow-up, analysis (a diagram is strongly recommended) |  |  |
|  |  | o Enrollment: the numbers of participants screened for eligibility, found to be eligible or not eligible, declined to be enrolled, and  enrolled in the study | 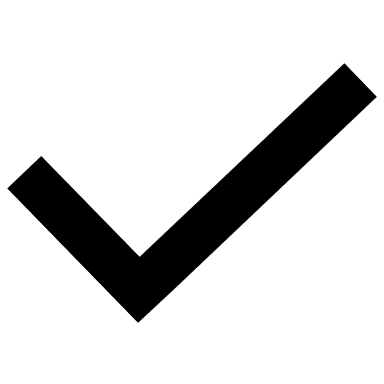 | **Results** paragraph 3, |
|  |  | o Assignment: the numbers of participants assigned to a study  condition | 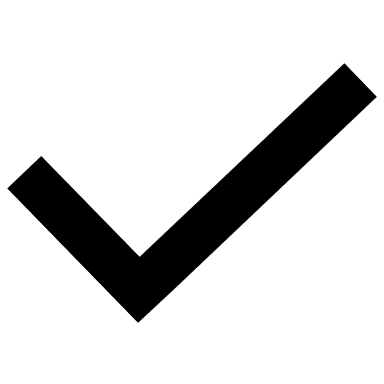 | **Results** paragraph 3 and paragraph 7 as well as Tables 1, 2 and 3 |
|  |  | o Allocation and intervention exposure: the number of participants assigned to each study condition and the number of participants  who received each intervention | 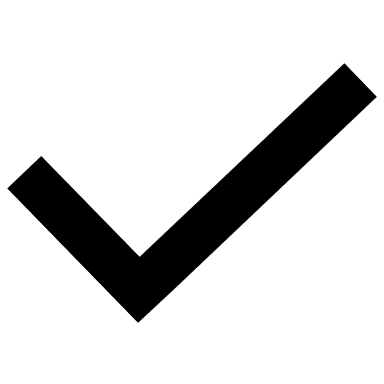 | **Results** paragraph 3 and paragraph 7 as well as Tables 1, 2 and 3 |
|  |  | o Follow-up: the number of participants who completed the follow- up or did not complete the follow-up (i.e., lost to follow-up), by  study condition | 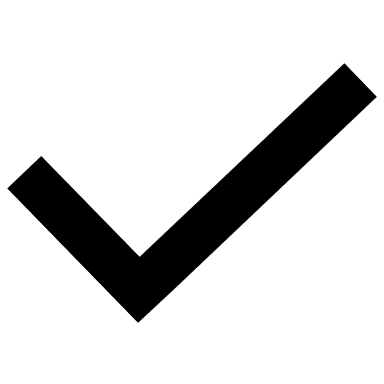 | **Results** paragraph 4 – last sentence and paragraph 7 |
|  |  | o Analysis: the number of participants included in or excluded from  the main analysis, by study condition | 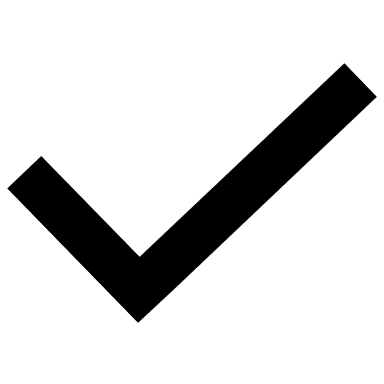 | **Results** paragraph 4 and paragraph 7 |
|  |  | 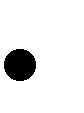 Description of protocol deviations from study as planned, along with reasons | N/A | Not applicable to our study |
| Recruitment | 13 | Dates defining the periods of recruitment and follow-up | 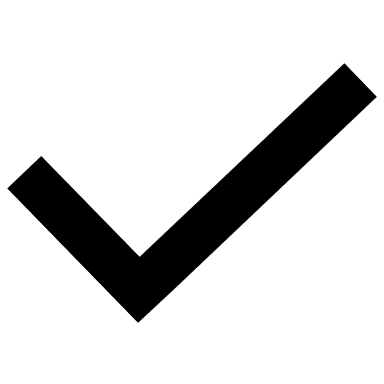 | **Results** paragraph 2 |
| Baseline Data | 14 | Baseline demographic and clinical characteristics of participants in each study condition | N/A | Not applicable to our study |
|  |  | Baseline characteristics for each study condition relevant to specific disease prevention research | 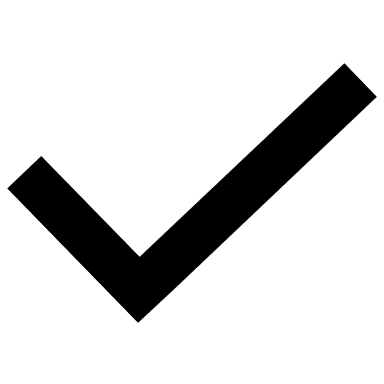 | **Results** Table 4 |
|  |  | Baseline comparisons of those lost to follow-up and those retained, overall and by study condition | N/A | Not applicable to our study |
|  |  | Comparison between study population at baseline and target population of interest | 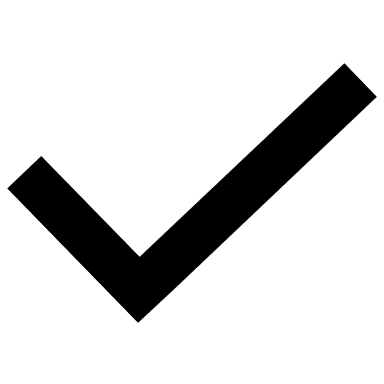 | **Results** Table 4 and 5 and paragraph 10- |
| Baseline equivalence | 15 | 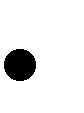 Data on study group equivalence at baseline and statistical methods used to control for baseline differences | 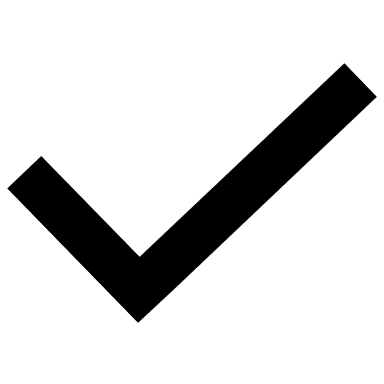 | **Results** paragraphs 9 and 10 |


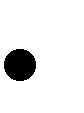

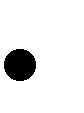

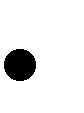


| Numbers analyzed | 16 | 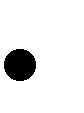 Number of participants (denominator) included in each analysis for each study condition, particularly when the denominators change for different  outcomes; statement of the results in absolute numbers when feasible | 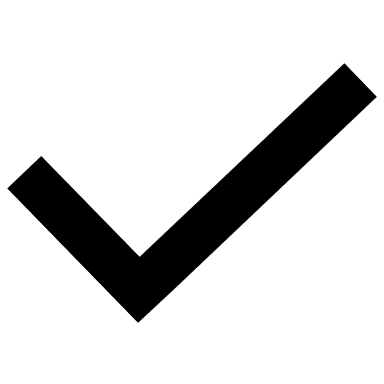 | **Results** paragraph 9 discusses the intervention population contribution to overall notifications |
| --- | --- | --- | --- | --- |
|  |  | 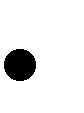 Indication of whether the analysis strategy was “intention to treat” or, if not, description of how non-compliers were treated in the analyses | N/A | N/A to our study since its goal was to identify people with TB and HIV and provide access to treatment |
| Outcomes and estimation | 17 | 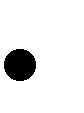 For each primary and secondary outcome, a summary of results for each estimation study condition, and the estimated effect size and a confidence interval to indicate the precision | 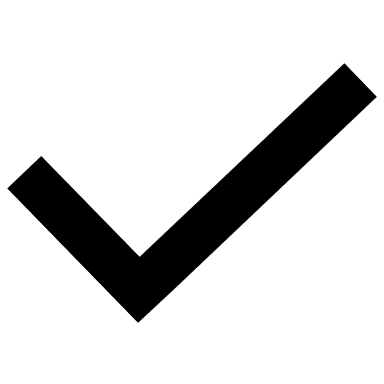 | Table 3 for HIV, Table 6 for TB |
|  |  | 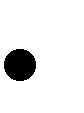 Inclusion of null and negative findings | N/A | Not applicable to our study |
|  |  | Inclusion of results from testing pre-specified causal pathways through which the intervention was intended to operate, if any | N/A | Not applicable to our study |
| Ancillary  analyses | 18 | 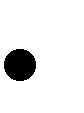 Summary of other analyses performed, including subgroup or restricted analyses, indicating which are pre-specified or exploratory | 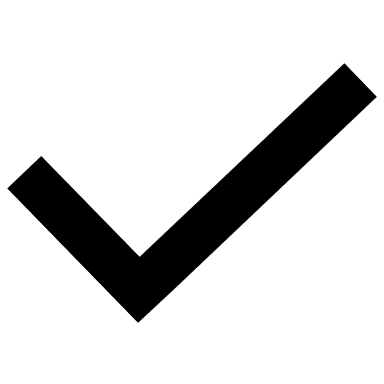 | Tables 4, 5 and 6 in **Results** signify all the analysis conducted |
| Adverse events | 19 | 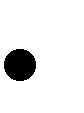 Summary of all important adverse events or unintended effects in each study condition (including summary measures, effect size estimates, and  confidence intervals) | N/A | Not applicable to our study |
| **DISCUSSION** | | | | |
| Interpretation | 20 | 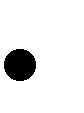 Interpretation of the results, taking into account study hypotheses, sources of potential bias, imprecision of measures, multiplicative analyses,  and other limitations or weaknesses of the study | 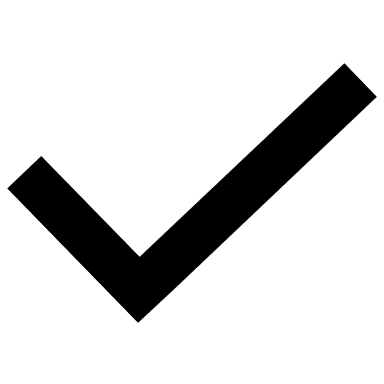 | **Discussion**, paragraph 1, paragraph 2 |
|  |  | Discussion of results taking into account the mechanism by which the intervention was intended to work (causal pathways) or alternative mechanisms or explanations | 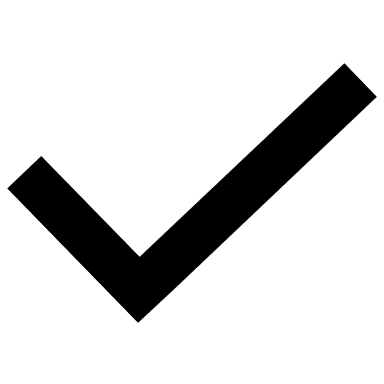 | **Discussion**, paragraph 1-2, and paragraph 3 and 4 |
|  |  | Discussion of the success of and barriers to implementing the intervention, fidelity of implementation | 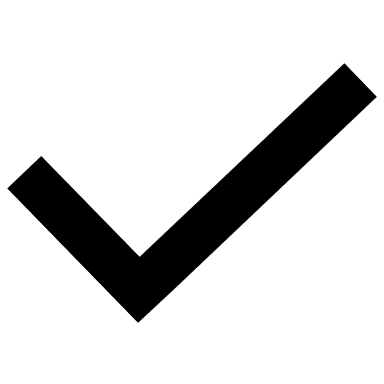 | **Discussion** – barriers and fidelity discussed throughout |
|  |  | Discussion of research, programmatic, or policy implications | 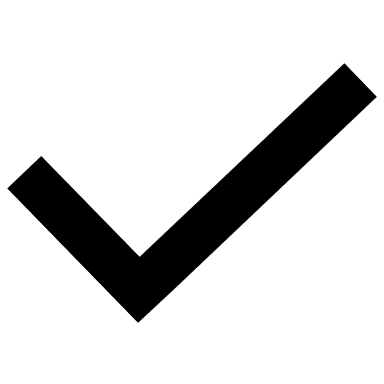 | **Discussion**, paragraph 1-2, and paragraph 3 and 4 |
| Generalizability | 21 | 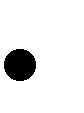 Generalizability (external validity) of the trial findings, taking into account the study population, the characteristics of the intervention, length of follow-up, incentives, compliance rates, specific sites/settings involved in  the study, and other contextual issues | 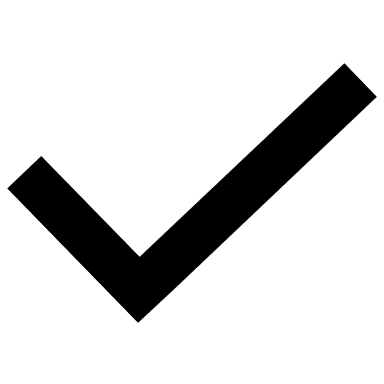 | **Discussion** paragraph 5/Limitations |
| Overall  Evidence | 22 | 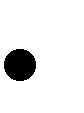 General interpretation of the results in the context of current evidence and current theory | 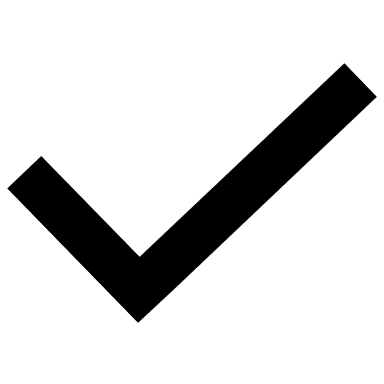 | **Discussion** paragraph 1 for TB and paragraph 2 for HIV |


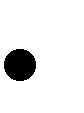


*From:* Des Jarlais, D. C., Lyles, C., Crepaz, N., & the Trend Group (2004). Improving the reporting quality of nonrandomized evaluations of behavioral and public health interventions: The TREND statement. *American Journal of Public Health*, 94, 361-366. For more information, visit: <http://www.cdc.gov/trendstatement/>
